# Exclusively Chemoselective *S*‑Acylation for Peptide Radiolabeling Using [^18^F]Fluoronicotinic Acid 4‑Nitrophenyl Ester as a Prosthetic Compound

**DOI:** 10.1021/acsomega.6c00097

**Published:** 2026-06-11

**Authors:** Nelson Nwaenie, Tuomas Karskela, Pyry Dillemuth, Johan Rajander, Pirjo Laakkonen, Anu J. Airaksinen, Xiang-Guo Li

**Affiliations:** † Turku PET Centre, 8058 University of Turku, Kiinamyllynkatu 4-8, Turku FI-20520, Finland; ‡ Department of Chemistry, University of Turku, Henrikinkatu 2, Turku FI-20500, Finland; § Turku Centre for Chemical and Molecular Analytics (CCMA), Åbo Akademi University, Henrikinkatu 2, Turku FI-20500, Finland; ∥ Accelerator Laboratory, Åbo Akademi University, Kiinamyllynkatu 4-8, Turku FI-20520, Finland; ⊥ Translational Cancer Medicine Research Program, Faculty of Medicine, 3835 University of Helsinki, Haartmaninkatu 8, Helsinki FI-00014, Finland; # iCAN Flagship Program, University of Helsinki, Haartmaninkatu 8, Helsinki FI-00014, Finland; ∇ Laboratory Animal Centre, HiLIFE University of Helsinki, Haartmaninkatu 8, Helsinki FI-00014, Finland; ○ Turku PET Centre, Turku University Hospital, Kiinamyllynkatu 4-8, Turku FI-20520, Finland; ◆ InFLAMES Research Flagship, University of Turku, Tykistökatu 6, Turku FI-20520, Finland

## Abstract

Sulfhydryl functionality is useful for the chemoselective and site-specific conjugation of biomolecules for medical diagnosis and therapeutic purposes. Several types of thiol-reactive conjugation chemistry have been developed, including a maleimide-based addition reaction. Our previous study showed that activated 6-[^18^F]­fluoronicotinic acid ([^18^F]­FNA) ester chemoselectively forms an *S*-acylated product with peptide ACooP (H-ACGLSGLGVA-NH_2_) bearing both a free amino group and a sulfhydryl group for positron emission tomography applications. The aim of this work is to better understand the chemoselectivity of *S*-acylation and explore its potential use for site-specific peptide radiolabeling. Accordingly, three new peptide variants of the decapeptide ACooP were designed as model sequences bearing both free amino and sulfhydryl functionalities, with the cysteine residue located at different positions in the sequences. The peptide variants C@3 (H-AGCLSGLGVA-NH_2_), C@4 (H-AGLCSGLGVA-NH_2_), and C@5 (H-AGLSCGLGVA-NH_2_) were conjugated with FNA to prepare the corresponding nonradioactive reference compounds. The conjugated products were characterized using one- and two-dimensional nuclear magnetic resonance analysis and high-resolution mass spectrometry. Peptide radiolabeling tests were performed using [^18^F]­FNA 4-nitrophenyl ester as the prosthetic group at pH 8.6 and 7.4. The radiolabeled products were *S*-acylated compounds with high or exclusive chemoselectivity (>95%) in all three cases. To demonstrate the utility of this type of chemoselective *S*-acylation for radiotracer preparation, [^18^F]­FNA-*S*-C@5 was prepared with a radiochemical purity of 97.0% ± 0.8 (n = 3) and a decay-corrected radiochemical yield of 16.1% ± 3.5. A batch size of the end product with hundreds of MBq was easily achieved, which is sufficient for PET imaging applications. [^18^F]­FNA-*S*-C@5 showed limited in vitro stability in rat plasma, with intact tracer observed at 13.1% ± 4.2 (n = 3) after 15 min of incubation. In conclusion, chemoselective *S*-acylation-based peptide radiolabeling was achieved using [^18^F]­FNA 4-nitrophenyl ester, and this reaction holds promise for highly chemoselective and site-specific radiolabeling of other types of biomolecules.

## Graphical abstract

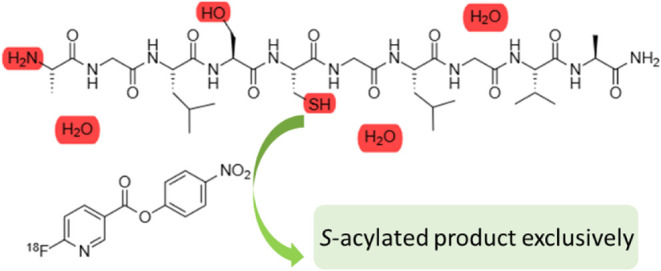

## Introduction

Radiolabeled peptides and peptidomimetics are an important class of radiotheranostics in nuclear medicine for precision cancer care.^1,2^ Prosthetic compounds are often needed for conjugation to radiolabel peptides based on different types of chemistry. One such prosthetic compound is fluorine-18 (^18^F)-labeled 6-fluoronicotinic acid ([^18^F]­FNA),^3^ which has proven useful in the clinical setting for positron emission tomography (PET) applications.^4,5^ [^18^F]­FNA is stable *in vivo* and defluorination is not significant (if any).^6^ In practice, [^18^F]­FNA is conjugated to peptides and biomolecules via activated esters, including the [^18^F]­FNA 4-nitrophenyl ester (Figure 1). The esters of [^18^F]­FNA can be conveniently produced with one-step radiosynthesis, even with on-resin^18^F-fluorination, omitting the conventional azeotropic drying process for [^18^F]­fluoride.^7^ Thus, far, [^18^F]­FNA has been used as a prosthetic group for radiolabeling various molecules, including peptides and antibody fragments.^3,8,9^

**Figure 1:**
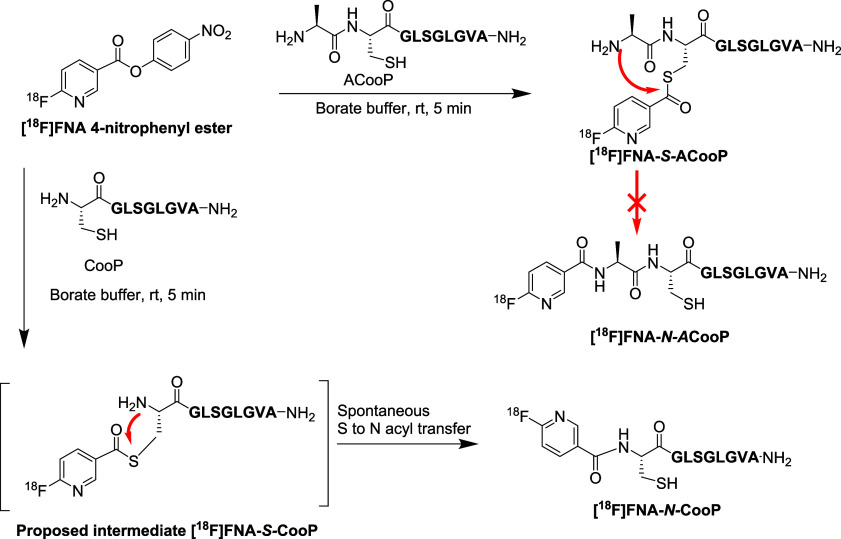
Proposed mechanism of chemoselective acylation of cysteine-containing peptides. The radiosynthesis of [^18^F]­FNA-*N*-CooP (C@1) and [^18^F]­FNA-*S*-ACooP (C@2) has been previously published.^10,11^

[^18^F]­FNA esters have been conventionally used as *N*-acylation agents that react with amino-functionalized biomolecules. However, we have recently found that [^18^F]­FNA 4-nitrophenyl ester can acylate not only amino groups but also sulfhydryl groups, and the acylation is exclusively chemoselective and reacts rapidly under mild conditions.^10,11^ The conjugation reaction between decapeptide ACooP (sequence H-ACGLSGLGVA-NH_2_, also named C@2 to indicate the location of the cysteine residue in the sequence) and [^18^F]­FNA 4-nitrophenyl ester was accomplished within 10 min in borate buffer (pH 8.6) at room temperature (r.t.), affording the formation of *S*-acylated product [^18^F]­FNA-*S*-ACooP (Figure 1).^10^ When using the CooP peptide (sequence H-CGLSGLGVA-NH_2_, also named C@1) under similar reaction conditions, we obtained the *N*-acylated product [^18^F]­FNA-*N*-CooP as the sole product.^11^ ACooP and CooP are peptide ligands that target fatty acid binding protein 3 (FABP3) in brain tumors.^12,13^ Both ACooP and CooP have free amino and sulfhydryl groups in their sequences, and the chemoselectivity was exclusively high (>95%) in both cases. Chemoselective radiolabeling holds promise in the development of new radiopharmaceuticals and biomaterials without the need for time-consuming protection and deprotection processes.^14,15^ In addition, chemoselective reactions may facilitate fully automated production of radiopharmaceuticals, as each single chemical transformation step poses extra challenges in process engineering and instrumentation.

Sulfhydryl functionality provides a unique opportunity for site-specific conjugation of biomolecules, including peptides and antibodies, as cysteine residues are commonly present in natural proteins or peptides. Furthermore, many biomolecules have been intentionally engineered to bear a sulfhydryl group for site-specific conjugation for different applications, including antibody–drug conjugates.^16^ Accordingly, the development of new methods for thiol-reactive reactions is an ever-continuing endeavor in the research community.^14,16^ The aim of this work is to better understand the mechanisms underlying chemoselective *S*-acylation and explore its application for site-specific peptide radiolabeling. Comparing the sequences of ACooP and CooP, the cysteine residue is located either at the *N*-terminus or next to it. We hypothesized that acylation occurs initially at the sulfhydryl group of the cysteine residue, which may be followed by an intramolecular *S*-to-*N* acyl transfer reaction. This likely occurs when a five-membered ring transition state is formed, as in the case of [^18^F]­FNA-*N*-CooP (Figure 1). In contrast to [^18^F]­FNA-*S*-ACooP, the corresponding transition state would have an unfavorable eight-membered ring, and the *S*-to-*N* acyl transfer does not occur under the experimental conditions used. A long-range *S*-to-*N* acyl transfer may also occur under suitable conditions; however, five-membered transition state rings are usually most favored.^17,18^ Herein, we report our evidence supporting this hypothesis, and the studies were performed with three new peptide sequences, namely, H-AGCLSGLGVA-NH_2_ (C@3), H-AGLCSGLGVA-NH_2_ (C@4), and H-AGLSCGLGVA-NH_2_ (C@5), with the cysteine residue at different positions.

## Results and Discussion

### Study Design

To test the above-mentioned hypothesis, we designed three new peptide sequences, named C@3, C@4, and C@5, by sequentially inserting the cysteine residue. According to the proposed mechanism shown in Figure 1, *S*-acylated products are formed in the conjugation of C@3, C@4, and C@5 with [^18^F]­FNA 4-nitrophenyl ester. In the three peptide sequences, the cysteine’s sulfhydryl functionality becomes increasingly distant from the *N*-terminus amino group, and we anticipate that *S*-to-*N* acyl transfer will not occur. Peptides C@3, C@4, and C@5 are also used as model sequences to demonstrate the potential application for biomolecule site-specific radiolabeling. The peptides C@3, C@4, and C@5 were custom-synthesized from United Biosystems (USA) and used in the conjugation experiments as described below.

### Preparation and Characterization of FNA-*S*-C@3, FNA-*S*-C@4, and FNA-*S*-C@5

The next step was to conduct conjugation reactions of the three peptides with the nonradioactive prosthetic compound FNA 4-nitrophenyl ester. Similar to the published reaction conditions for FNA-*S*-ACooP (FNA-*S*-C@2) preparation,^10^ FNA-*S*-C@3 was prepared in a reaction mixture of C@3 and FNA 4-nitrophenyl ester in borate buffer (pH 8.6) at r.t. In the nonradioactive synthesis, the prosthetic compound FNA 4-nitrophenyl ester was used 3.2-fold to the amount of peptide C@3. After 10 min of reaction, the product FNA-*S*-C@3 was isolated by semipreparative high-performance liquid chromatography (HPLC) on a reversed-phase HPLC column, and solvents were removed. FNA-*S*-C@3 was obtained as a solid in 15.3% isolated yield. Following similar experimental procedures, FNA-*S*-C@4 and FNA-*S*-C@5 were obtained in isolated yields of 16.9% and 18.6%, respectively.

To characterize the chemical structures of the obtained compounds, nuclear magnetic resonance (NMR) analysis and high-resolution mass spectrometry (MS) were performed. For FNA-*S*-C@3, the protons of cysteine were identified at the following chemical shifts (in ppm) in one-dimensional (1D) NMR: 3.31 and 3.48 (Cysβ), 4.64 (Cysα), and 8.41 (CysNH), as shown in Figure 2. The carbonyl carbon of FNA was observed at 189.01 ppm. All chemical shifts were identified using two-dimensional (2D) NMR analysis (Figures S1–S3), including total correlation spectroscopy (TOCSY) and heteronuclear single quantum coherence (HSQC) spectra, with further confirmation from 2D heteronuclear multiple bond correlation (HMBC) spectra. HMBC elucidated the sequence by the interaction of backbone amide protons with the neighboring carbonyl carbon. According to the NMR analyses (Figures S4–S7) for FNA-*S*-C@4, cysteine protons were identified at the following chemical shifts (in ppm): 3.32 and 3.52 (Cysβ), 4.60 (Cysα), and 8.41 (CysNH). The carbonyl carbon of FNA was observed at 188.97 ppm. For FNA-*S*-C@5 (Figures S8–S11), cysteine protons were identified at the following chemical shifts (in ppm): 3.31 and 3.56 (Cysβ), 4.56 (Cysα), and 8.23 (CysNH). The carbonyl carbon of FNA was observed at 189.01 ppm. These chemical shifts are expected for cysteine thioesters. If the cysteine sulfhydryl group was free, the Cysβ proton chemical shifts would be at approximately 2.7–2.8 ppm and Cysα at 4.4 ppm. Moreover, the chemical shift of FNA carbonyl carbon would be approximately 163 ppm for an amide bond. The sulfhydryl proton was not detected in the spectra. The FNA carbonyl carbon is consistent with a thioester structure. Furthermore, the exact mass of the products was analyzed with the liquid chromatography-electrospray ionization-mass spectrometry/mass spectrometry (LC-ESI-MS/MS, Figures 3, S12, and S13). Taken together, FNA-*S*-C@3, FNA-*S*-C@4, and FNA-*S*-C@5 are *S*-acylated products. In addition to the anticipated products, we attempted to isolate the side products from the reaction mixtures. However, the quantity of isolated side products was insufficient for reliable chemical identity analysis. Because of the observations of side products under ultraviolet (UV) detection and relatively low isolated chemical yields (<20%), we cannot conclude that the reactions were chemoselective toward *S*-acylation in these nonradioactive conjugation experimental settings. To make this clear, we have summarized the reaction conditions and observed products under nonradioactive and radioactive conjugation experiments in Supplementary Table S1. The purpose of nonradioactive conjugation was to prepare reference compounds that can be used to confirm the chemical identity of radioactive products. Chemoselective acylation in a nonradioactive experimental setting was not the study objective.

**Figure 2:**
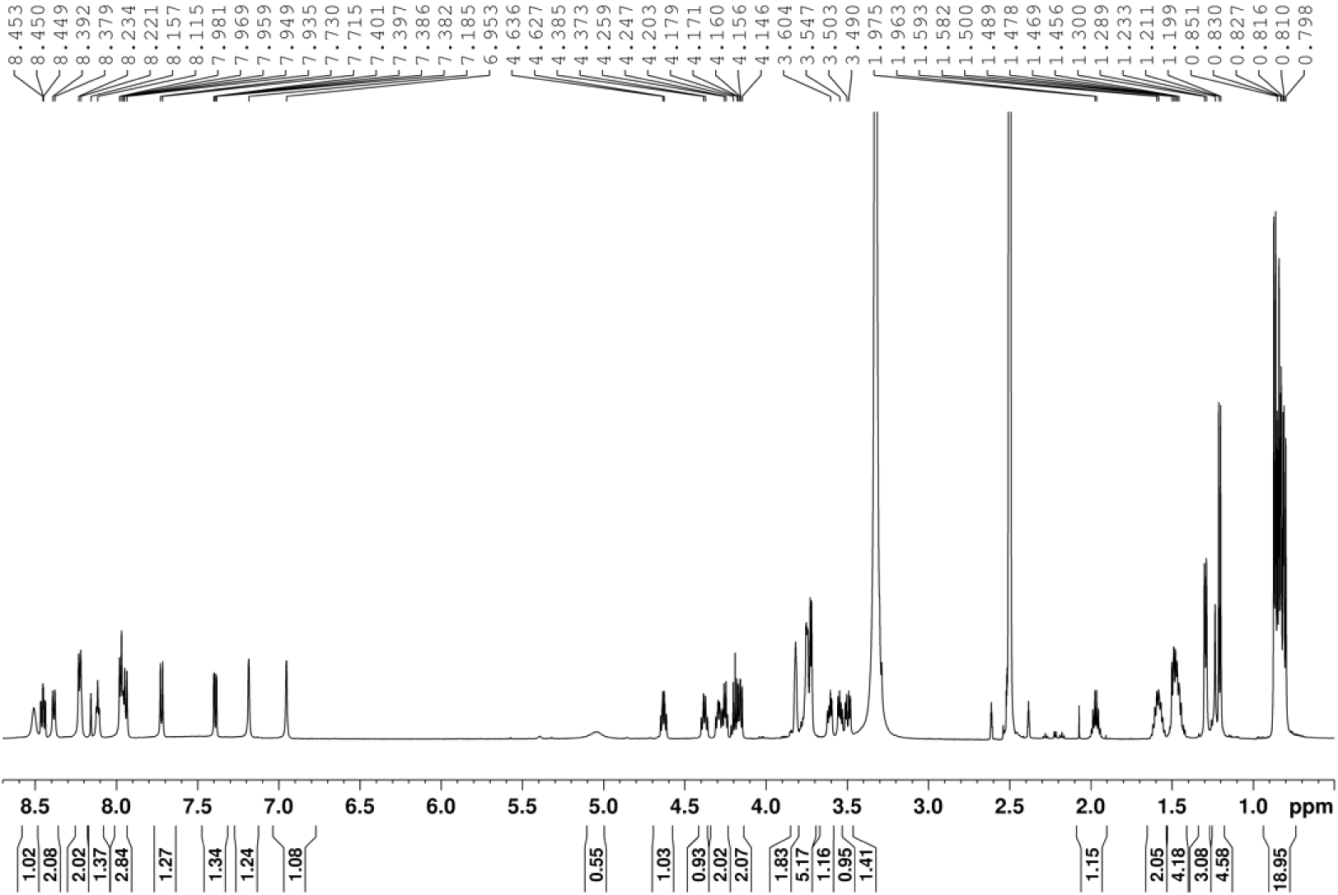
^1^H NMR spectrum of FNA-*S*-C@3.

**Figure 3:**
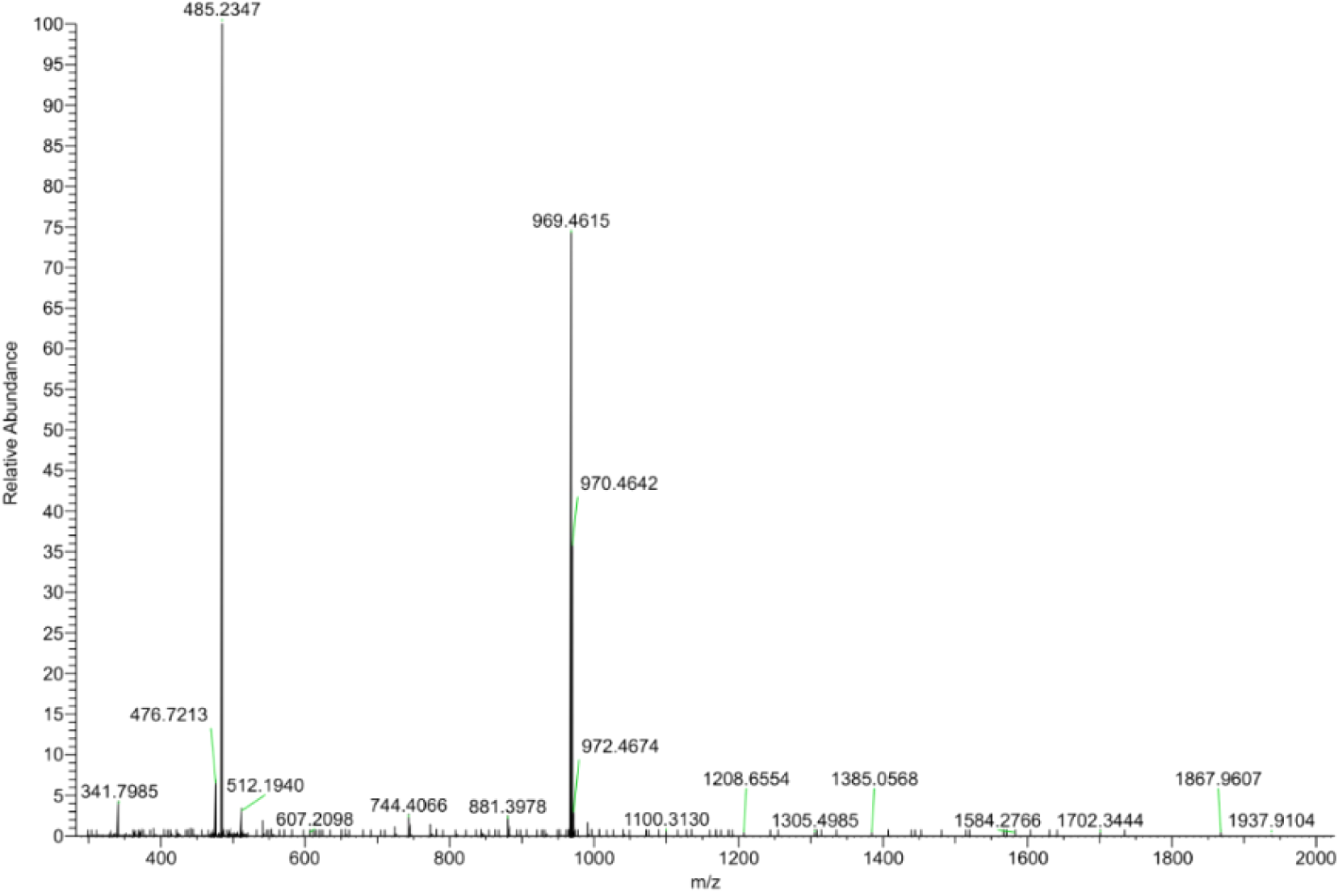
MS spectrum of FNA-*S*-C@3.

Taken together, according to the mass spectra the main products had one FNA group attached to the peptide chain. Cysteine side chain proton chemical shifts and FNA carbonyl carbon chemical shifts showed that FNA was bonded to the cysteine residues by a thioester linkage, while chemical shifts that would reveal amide bonds at the *N*-terminal alanine or an unsubstituted cysteine residue were missing. These data ruled out that the products were *N*-acylated.

### Peptide Radiolabeling Is Highly Chemoselective toward *S*-Acylation

To study chemoselectivity under radiolabeling conditions, the peptides C@3, C@4, and C@5 were subjected to conjugation reactions in the presence of [^18^F]­FNA 4-nitrophenyl ester in borate buffer (pH 8.6) at r.t. In the radiolabeling experiments, peptide precursors were used at a concentration of 8.9 mM, which was in large excess in relation to the molar amount of the prosthetic compound [^18^F]­FNA 4-nitrophenyl ester (<20 μM in typical cases). A large excess of peptides was used to drive the reaction to completion within 10 min, which is desirable for radiopharmaceutical production with relatively shorter-lived radionuclides in the clinical setting. At 10 min of reaction, samples were taken from the reaction mixtures without any purification and analyzed with HPLC equipped with both radioactivity and UV detectors, and the chemical identity of the radioactive products was confirmed with HPLC analysis of the corresponding nonreference compounds (Figure 4). In the radiolabeling reactions of [^18^F]­FNA-*S*-C@3 and [^18^F]­FNA-*S*-C@5, the anticipated products were the radioactive products in predominant amounts (>95%, Figure 4A,E); in the radiolabeling reaction of [^18^F]­FNA-*S*-C@4, the anticipated product was the only radioactive product (>99%, Figure 4C). In all three cases, the chemical identity of the products was confirmed by coinjection of the radioactive samples with the nonradioactive reference compounds FNA-*S*-C@3, FNA-*S*-C@4, and FNA-*S*-C@5, as prepared above. These results confirmed that the reactions were highly or exclusively chemoselective toward the sulfhydryl functionality under the experimental radiolabeling conditions. Furthermore, all three reactions proceeded rapidly (maximum 10 min at r.t.) and the amount of unreacted prosthetic compound [^18^F]­FNA 4-nitrophenyl ester was not detectable. Control HPLC analysis of the [^18^F]­FNA 4-nitrophenyl ester standard was performed, indicating that the retention time of [^18^F]­FNA 4-nitrophenyl ester was 14.88 min (Figure 4G). Taken together with the published results for chemoselective radiosynthesis of [^18^F]­FNA-*N*-C@1^11^ and [^18^F]­FNA-*S*-C@2^10^, it was evident that intramolecular *S*-to-*N* acyl transfer did not occur in cases where the cysteine residue was not the first amino acid residue in the peptide sequence (Figure 5). This was assumed to be due to the unfavorable ring sizes of 11, 14, and 17 in the [^18^F]­FNA-*S*-C@3, [^18^F]­FNA-*S*-C@4, and [^18^F]­FNA-*S*-C@5 transition states. Therefore, our hypothesis was proven. However, we must emphasize that the proposed reaction mechanism was based on the observation of conjugation products instead of reaction intermediates. The above radiolabeling tests were performed with a small amount of radioactivity (approximately 10 MBq), and the end product was not purified. To demonstrate the use of this type of *S*-acylation reaction for potential PET imaging applications, we produced three batches of [^18^F]­FNA-*S*-C@5 and purified the end product using semipreparative HPLC. The radioactivity of the end product was 361.3 ± 71.6 MBq (n = 3) starting from 7.5 ± 1.0 GBq of [^18^F]­fluoride. Further scale-up radiosynthesis was not necessary, keeping in mind the as low as reasonably achievable (ALARA) principle in radiation work. The total synthesis time was 188.0 ± 15.1 min. The decay-corrected radiochemical yield was 16.1% ± 3.5, and the radiochemical purity was 97.0% ± 0.8. The molar activity of [^18^F]­FNA-*S*-C@5 was 66.7 GBq/μmol.

**Figure 4:**
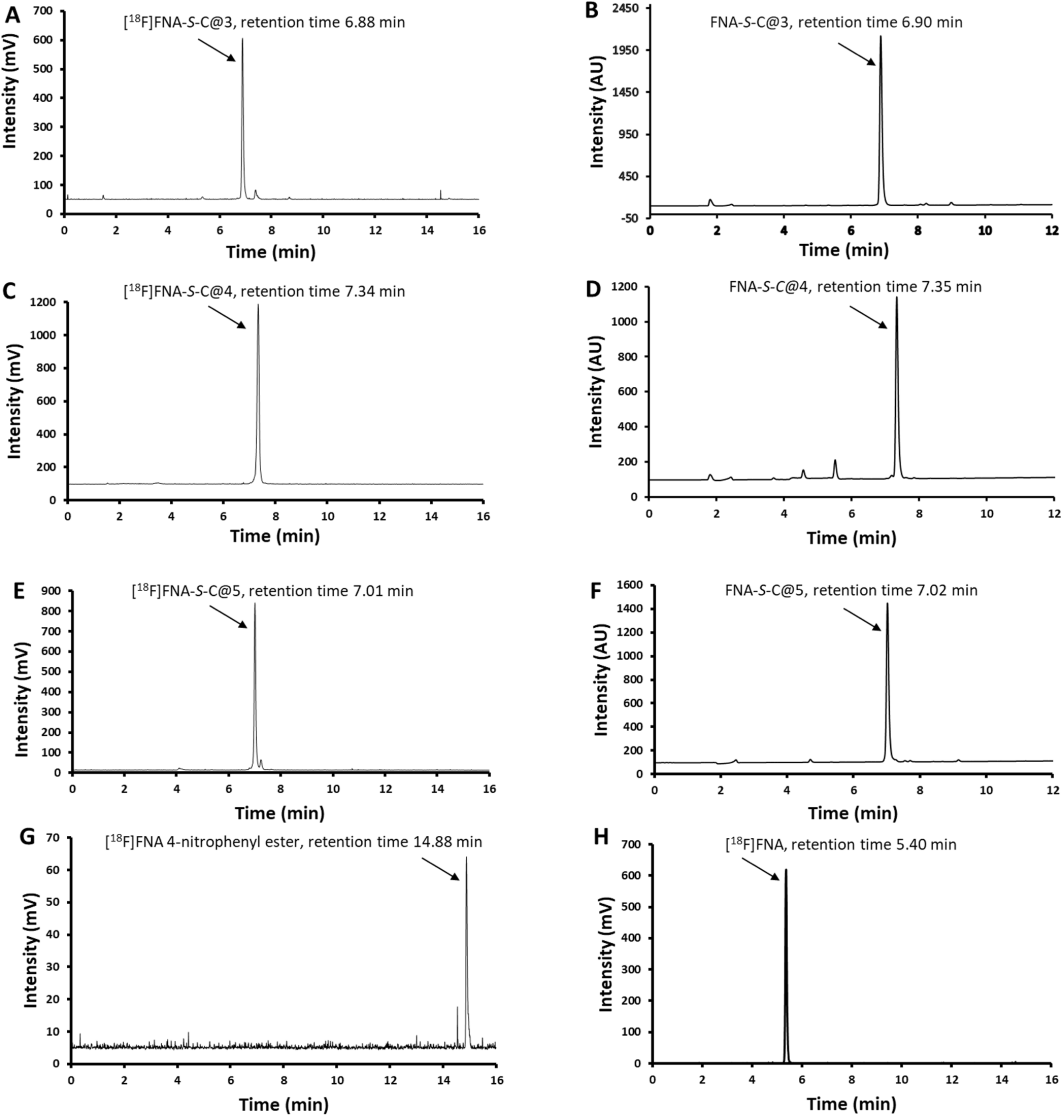
HPLC chromatograms of peptide radiolabeling tests and nonradioactive reference compounds. (A), (C), and (E) are samples from conjugation reaction mixtures of C@3, C@4, and C@5 with [^18^F]­FNA 4-nitrophenyl ester at pH 8.6 for 10 min at r.t., respectively. (B), (D), and (F) are nonradioactive reference compounds of FNA-*S*-C@3, FNA-*S*-C@4, and FNA-*S*-C@5, respectively. (G) was the reference compound of the prosthetic compound [^18^F]­FNA 4-nitrophenyl ester. (H) was the reference compound of [^18^F]­FNA.

**Figure 5:**
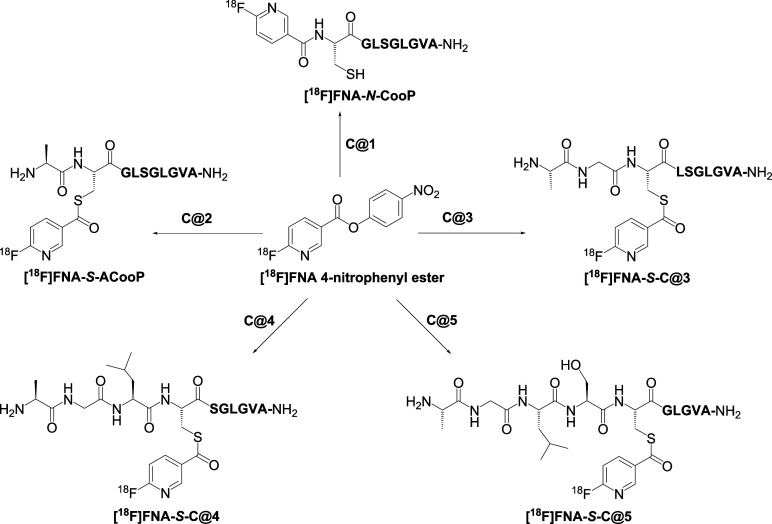
Graphic summary of chemoselective peptide acylation using [^18^F]­FNA 4-nitrophenyl ester as the prosthetic group. Among the five peptides, C@1 and C@2 have been published previously.^10,11^

### Exclusively Chemoselective Radiolabeling at Physiological pH

For radiolabeling applications of large biomolecules (e.g., intact antibodies), performing conjugation reactions at physiological pH is desirable to reduce the risk of damaging the biomolecules. This prompted us to test whether radiolabeling could still occur and would still be chemoselective in a phosphate buffer at pH 7.4. Accordingly, peptide precursors C@3, C@4, and C@5 were subjected to conjugation reactions similar to those described above, except that phosphate buffer (pH 7.4) was used as the reaction medium. According to the HPLC analyses of samples from the reaction mixtures without any purification, the *S*-acylated product was the sole radiolabeled product in each case (Figure 6). Reactions completed within 10 min at r.t. The chemical identity of the radiolabeled products was confirmed by HPLC analysis using the nonradioactive reference compounds. This result indicates that [^18^F]­FNA 4-nitrophenyl ester holds promise for radiolabeling biomolecules at physiological pH and mild reaction conditions.

**Figure 6:**
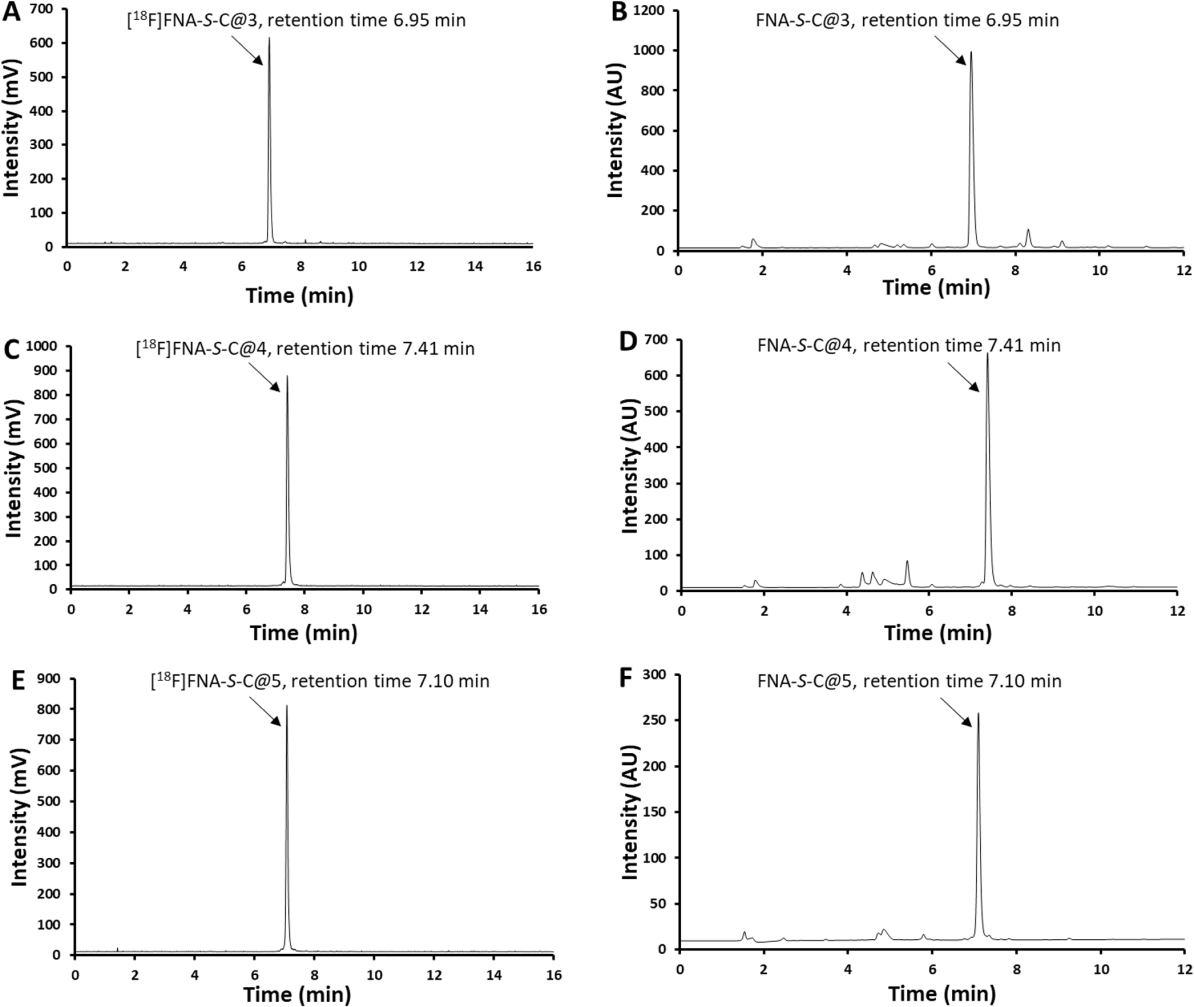
HPLC chromatograms of peptide radiolabeling tests and nonradioactive reference compounds. (A), (C), and (E) are samples from conjugation reaction mixtures of C@3, C@4, and C@5 with [^18^F]­FNA 4-nitrophenyl ester at pH 7.4 for 10 min at r.t., respectively. (B), (D), and (F) are nonradioactive reference compounds of FNA-*S*-C@3, FNA-*S*-C@4, and FNA-*S*-C@5, respectively.

### The Sulfhydryl Group Is the Most Competitive Nucleophile

The clean reaction profiles in the above-mentioned radiolabeling experiments indicate that the cysteine sulfhydryl group is the most competitive nucleophile under those reaction conditions. Potentially competing nucleophiles exist, as shown with C@5 as an example (Figure 7). In addition to the amino and hydroxyl groups in the peptides, water molecules in the reaction media are in even larger molar excess in relation to the acyl donor [^18^F]­FNA 4-nitrophenyl ester. However, the hydrolytic product [^18^F]­FNA was not observed in any of the conjugation reactions, according to the control tests in HPLC analysis using [^18^F]­FNA as the reference compound (Figure 4H). This result showed that [^18^F]­FNA 4-nitrophenyl ester not only has high enough reactivity for *S*-acylation but also sufficient resistance to hydrolysis as a side reaction. These properties make [^18^F]­FNA 4-nitrophenyl ester a favorable prosthetic compound for radiolabeling biomolecules. Conventionally, [^18^F]­FNA esters are supposed to have *N*-acylation of amino groups in proteins. From the results presented in this work, it is possible that free thiol groups may also be acylated. Therefore, we posit that the nature of conjugation chemistry should be confirmed in cases where the proteins have both free amino and thiol groups in their sequences.

**Figure 7:**
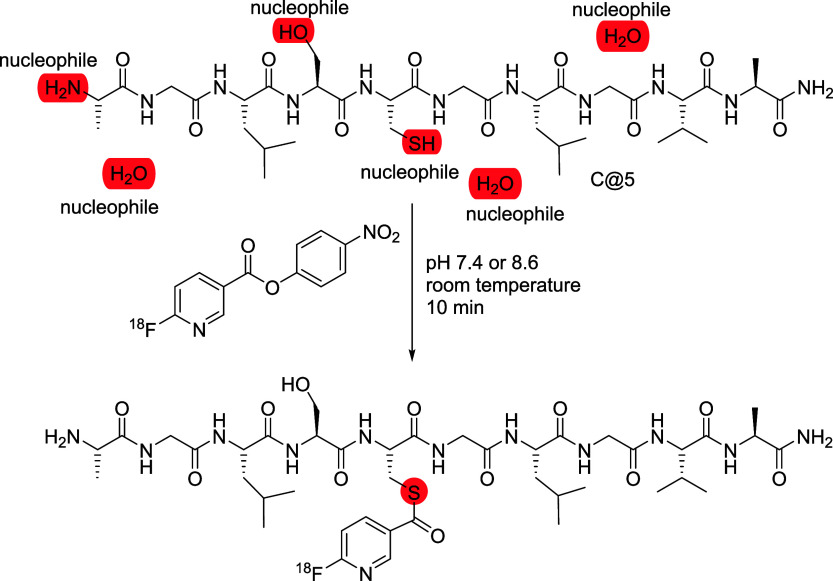
Competing nucleophiles (marked in red) in the conjugation of peptide C@5 with [^18^F]­FNA 4-nitrophenyl ester.

### In Vitro Stability Tests in Rat Plasma

As described above, the radiolabeled peptide [^18^F]­FNA-*S*-C@5 was selected to demonstrate the whole radiosynthesis procedure including end product isolation and formulation. To evaluate the stability of [^18^F]­FNA-*S*-C@5 in vitro, the tracer was mixed with rat plasma samples and incubated at 37 °C. Samples were taken at 5 and 15 min of incubation, and plasma proteins were removed and the supernatant samples were then analyzed by HPLC, using a [^18^F]­FNA-*S*-C@5 tracer standard as a reference for identification of intact tracer (Figure 8). The proportion of intact tracer in the plasma samples at 5 and 15 min of incubation was 43.9% ± 4.0 (n = 3) and 13.1% ± 4.2 (n = 3), respectively. Although a notable fraction of the tracer remained intact at 5 min of plasma incubation, rapid metabolism was observed with less than 15% remaining intact at 15 min. Several radiometabolites were detected, but their chemical identities were not investigated in this study. To further confirm the identity of the intact tracer in plasma samples, the tracer standard [^18^F]­FNA-*S*-C@5 was coinjected with a plasma supernatant sample collected at 15 min of incubation. A single peak at the expected retention time was observed. Taken together, [^18^F]­FNA-*S*-C@5 showed limited in vitro stability in rat plasma. Peptide instability has been well documented in the literature, and huge efforts have been devoted to the development of stabilization methods.^19^ The strategy for peptide stabilization needs to be developed case-by-case depending on the peptide sequences and their intended applications. This also holds true for methodological development for stabilization of [^18^F]­FNA-*S*-C@5.

**Figure 8:**
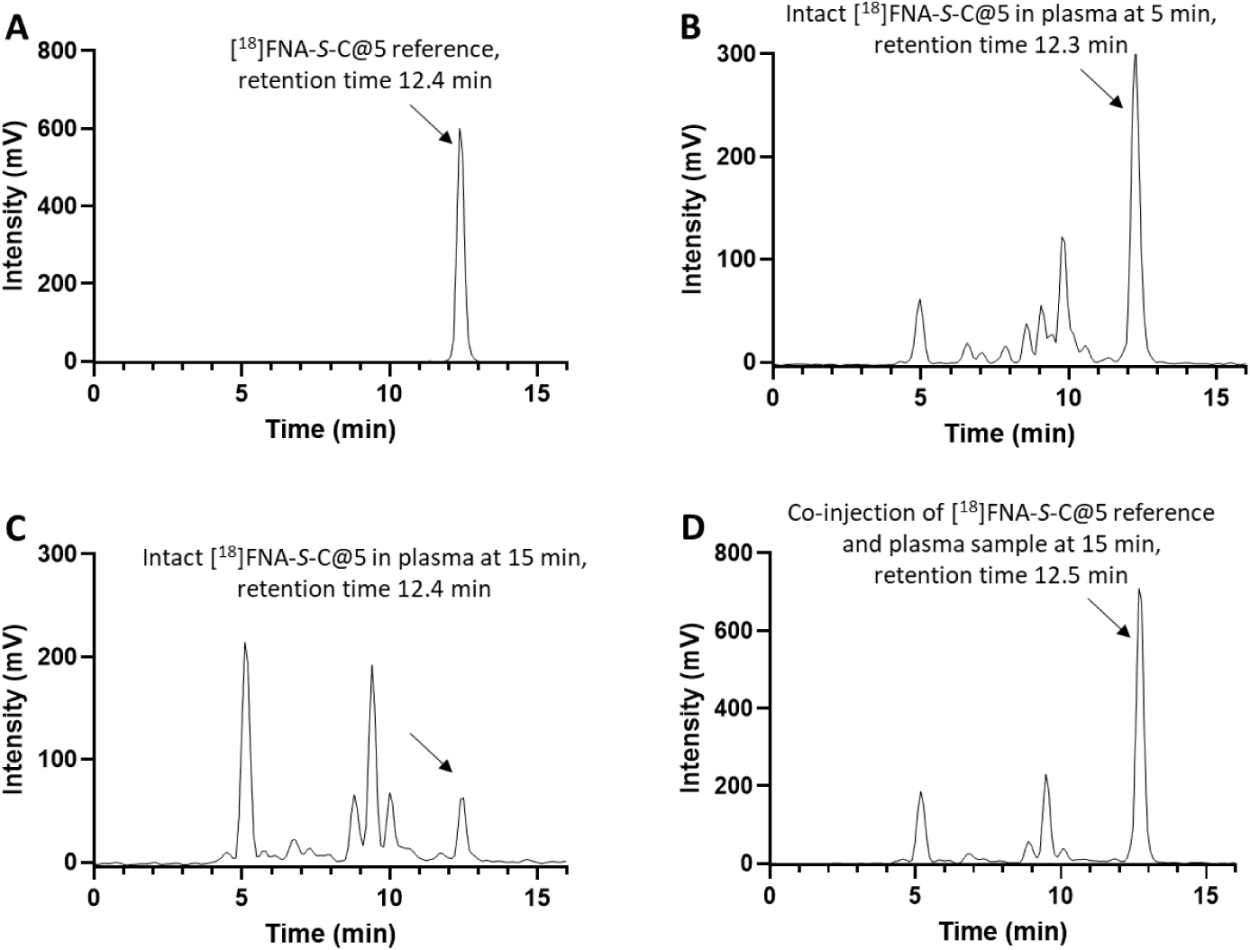
HPLC analysis of the in vitro stability of [^18^F]­FNA-*S*-C@5 in rat plasma with radioactivity detection. (A) Reference standard of [^18^F]­FNA-*S*-C@5. (B) Representative HPLC chromatogram of plasma samples taken at 5 min of incubation. (C) Representative HPLC chromatogram of plasma samples taken at 15 min of incubation. (D) Co-injection of [^18^F]­FNA-*S*-C@5 standard with a plasma sample taken at 15 min of incubation, confirming the chemical identity of intact [^18^F]­FNA-*S*-C@5.

## Conclusions

In the radiolabeling of all four decapeptides with cysteine residues located at the second, third, fourth, and fifth positions, exclusive chemoselectivity toward *S*-acylation was observed using [^18^F]­FNA 4-nitrophenyl ester as an acyl donor. This proved our hypothesis that *S*-to-*N* acyl transfer could not occur when these peptide sequences lack favorable five-membered ring transition states. Furthermore, the radiolabeling reactions are rapid and neat. This work may contribute to the further development of radiolabeling methods that are highly chemoselective for different types of biomolecules under mild reaction conditions. As a study limitation, the current work only demonstrated chemoselective radiolabeling of short and linear peptides with a single cysteine residue in the sequences. Further study is warranted to clarify the chemoselectivity on larger peptides and proteins bearing multiple cysteine residues. The model peptide [^18^F]­FNA-*S*-C@5 had limited in vitro stability in rat plasma, which is a challenge to be addressed for PET applications.

## Methods

### Materials and General Information

The peptides H-AGCLSGLGVA-NH_2_ (C@3), H-AGLCSGLGVA-NH_2_ (C@4), and H-AGLSCGLGVA-NH_2_ (C@5) were purchased from United Biosystems (Herndon, VA, USA). FNA 4-nitrophenyl ester was purchased from Enamine (Kyiv, Ukraine). NMR spectra were recorded using a Bruker Avance III 600 MHz equipped with a liquid nitrogen-cooled Prodigy TCI probe or a Bruker Avance III 500 MHz equipped with a liquid nitrogen-cooled Prodigy BBO probe (Bruker, Billerica, MA, USA). Chemical shifts of ^1^H NMR were reported relative to the solvent residual proton signal of dimethyl sulfoxide (DMSO-*d6*: δ = 2.50 ppm). Chemical shifts of ^13^C NMR were reported relative to the solvent signal (DMSO-*d6*: δ = 39.52 ppm). Characterization was performed using 1D and 2D NMR methods. The MS analysis was performed using a Q Exactive HF mass spectrometer (Thermo Fisher Scientific) equipped with a nanoelectrospray ion source coupled to an Easy-nLC 1000 HPLC nanoflow system (Thermo Fisher Scientific). The samples were trapped in a column (particle size 100 μm, internal diameter 2 cm) and then separated in an analytical column (particle size 75 μm, internal diameter 15 cm). ReproSil-Pur 3 μm 120 Å C18-AQ packing material (Dr. Maisch HPLC GmbH, Ammerbuch-Entringen, Germany) was used to pack both columns internally. MS analyses were performed at the Turku Proteomics Facility supported by Biocenter Finland. The chemical synthesis of reference compounds FNA-*S*-C@3, FNA-*S*-C@4 and FNA-*S*-C@5, and radiolabeling of [^18^F]­FNA-*S*-C@5 followed similar procedures as previously reported.^10^

### Preparation of FNA-*S*-C@3

Part of the experimental procedures and chemical characterization data have been described in an academic thesis of Master of Science degree at the University of Turku, Finland.^20^ In a 1,500-μL Eppendorf tube, 40 μL of FNA 4-nitrophenyl ester (19.1 μmol, 5.0 mg) in acetonitrile, 40 μL of borate buffer (300 mM, pH 8.6), 150 μL of peptide C@3 (5.9 μmol, 5.0 mg) solution in water (Trace SELECT Honeywell), and 200 μL of acetonitrile were added. The solution was agitated and allowed to homogenize at r.t. for approximately 10 min. The solution was purified using a semipreparative HPLC system. The HPLC included a reversed-phase C18 column manufactured by Phenomenex (Torrance, CA, USA), with dimensions of 250 × 10 mm, particle size 4 μm, and pore size 90 Å. The solvent flow rate was set at 5 mL/min. Solutions A and B were prepared using 0.1% trifluoroacetic acid (TFA) in water and 0.1% TFA in acetonitrile, respectively. The HPLC elution gradient used for the analysis was 0–10.5 min from 25% B to 50% B, and 10.5–20.0 min from 50% B to 80% B. The retention time of the product was at 6.7 min. Subsequently, the product fractions were mixed and dried in a vacuum drier (Heto Hetovac VR-1 and CT 60E Vacuum Concentrator, Vacuum Model Avc001). After drying, a solid white product appeared. The obtained product (0.9 mg, 0.9 μmol, isolated yield of 15.3%) was characterized using MS and 1D and 2D NMR. The chemical shifts in 1D NMR were designated using 2D NMR data. The chemical structure was characterized as follows. δ ^1^H (600 MHz; DMSO-*d*6) 0.80 (3 H, br, Me­(Val)), 0.82 (3 H, d, Me­(Leu)), 0.83 (3 H, d, Me­(Leu)), 0.84 (3 H, br, Me­(Val)), 0.87 (6 H, d, Me­(Leu)), 1.20 (3 H, d, Me­(Ala2)), 1.23­(2 H, br, CH_2_(Leu 1β)), 1.30 (3 H, d, Me­(Ala1)), 1.48 (2 H, m, CH_2_(Leu 2β)), 1.58 (2 H, m, CH­(Leuγ)), 1.97 (1 H, m, CH­(Valβ)), 3.31 (1 H, d, CH_2_(Cysβ)), 3.48 (1 H, d, CH_2_(Cysβ)), 3.55 (1 H, dd, CH_2_(Serβ)), 3.6 (1 H, m, CH_2_(Serβ)), 3.74 (2 H, dd, CH­(Gly1α)), 3.75 (2 H, dd, CH­(Gly2α)), 3.76 (1 H, dd, CH­(Ala1α)), 3.81 (2 H, br, CH­(Gly 3α)), 4.15 (1 H, d, CH­(Valα)), 4.19 (1 H, m, CH­(Ala2α)), 4.25 (1 H, m, CH­(Serα)), 4.29 (1 H, m, CH­(Leu1α)), 4.38 (1 H, m, CH­(Leu 2α)), 4.64 (1 H, m, CH­(Cysα)), 5.05 (1 H, br, SerOH), 6.96 (1 H, s, CONH_2_(Ala2)), 7.20 (1 H, s, CONH_2_(Ala2)), 7.40 (1 H, dd, 5-H FNA), 7.74 (1 H, d, NH­(Val)), 7.78 (1 H, d, NH­(Gly 3)), 7.97 (1 H, d, NH­(Leu 1)), 7.99 (2 H, d, NH­(Ala 2)), 8.00 (2 H, m, NH­(Ala1)), 8.13 (1 H, t, NH­(Gly1)), 8.17 (1 H, m, NH­(Leu 2)), 8.24 (1 H, d, NH­(Gly 2)), 8.35 (1 H, d, NH­(Ser)), 8.41 (1 H, d, NH­(Cys)), 8.45 (1 H, dt, 4-H­(FNA)), 8.78 (1 H, d, 2-H­(FNA)). δ ^13^C (125 MHz; DMSO-*d*6) 17.69 Me­(Val), 18.00 Me­(Ala2), 18.16 Me­(Ala1), 19.13 Me­(Val), 21.56 Me­(Leu), 23.09 Me­(Leu), 24.02 Me­(Leu), 24.10 Me­(Leuγ), 28.96 Cβ­(Leu1), 30.47 Cβ­(Val), 30.80 Cβ­(Cys), 30.81 Cβ­(Cys), 40.63 Cβ­(Leu2), 41.78 Cα­(Gly), 42.10 Cα­(Gly), 47.84 Cα­(Ala1), 48.60 Cα­(Gly), 49.39 Cα­(Ala2), 51.03 Cα­(Leu1), 51.20 Cα­(Leu2), 51.62 Cα­(Cys), 55.15 Cα­(Ser), 57.50 Cα­(Val), 61.59 Cβ­(Ser), 110.46 (d, 5-C­(FNA)), 131.72 (d, 3-C­(FNA)), 141.24 (d, 4-C­(FNA)), 146.99 (d, 2-C­(FNA)), 164.78 (d, 6-C­(FNA)), 168.23 CO­(Gly), 168.29 CO­(Gly), 169.83 CO­(Gly), 169.91 CO­(Cys), 169.95 CO­(Ala2), 169.98 CO­(Ser), 170.09 CO­(Ala1), 171.14 CO­(Val), 171.34 CO­(Leu1), 171.43 CO­(Leu2), 189.01 CO­(FNA). MS *m*/*z*: [M + H]^+^ calculated for C_41_H_66_FN_12_O_12_S, 969.4622; found 969.4615.

### Preparation of FNA-*S*-C@4

Part of the experimental procedures and chemical characterization data have been described in an academic thesis of Master of Science degree at the University of Turku, Finland.^20^ Following a similar preparation procedure as for FNA-*S*-C@3, compound FNA-*S*-C@4 (1.0 mg, 1.0 μmol) was prepared as a white solid product in 16.9% isolated chemical yield. The retention time of FNA-*S*-C@4 was 6.0 min with the semipreparative HPLC method described above. The chemical structure was characterized as follows. δ ^1^H (600 MHz; DMSO-*d*6) 0.80 (3 H, d, Me­(Val)), 0.81 (3 H, d, Me­(Val)), 0.82 (3 H, d, Me­(Leu)), 0.84 (3 H, d, Me­(Leu)), 0.86 (6 H, d, Me­(Leu)), 1.21 (3 H, d, Me­(Ala2)), 1.34 (3 H, d, Me­(Ala1)), 1.45 (4 H, m, CH_2_(Leu 1, 2β)), 1.58 (2 H, m, CH­(Leuγ)), 1.97 (1 H, m, CH­(Valβ)), 3.32 (1 H, br, CH_2_(Cysβ)), 3.52 (1 H, m, CH_2_(Cysβ)), 3.55 (1 H, m, CH_2_(Serβ)), 3.62 (1 H, m, CH_2_(Serβ)), 3.74–3.83 (6 H, d, CH­(Glyα)), 3.87 (1 H, br, CH­(Ala1α)), 4.15 (1 H, d, CH­(Valα)), 4.20 (1 H, d, CH­(Ala2α)), 4.25 (1 H, m, CH­(Serα)), 4.27 (1 H, m, CH­(Leu1α)), 4.30 (1 H, m, CH­(Leu2α)), 4.60 (1 H, m, CH­(Cysα)), 5.05 (1 H, t, (SerOH)), 6.96 (1 H, s, CONH_2_(Ala2)), 7.20 (1 H, s, CONH_2_(Ala2)), 7.40 (1 H, dd, 5-H­(FNA)), 7.74 (1 H, d, NH­(Val)), 7.80 (2H, m, NH­(Ala 1)), 7.97 (1 H, d, NH­(Ser)), 8.00 (1 H, m, NH­(Leu1)), 8.17 (1 H, m, NH­(Gly1)), 8.19 (1 H, dd, NH­(Gly2)), 8.24 (1 H, dd, NH­(Gly3)), 8.36 (1 H, d, NH­(Leu 2)), 8.45­(1 H, dt, 4-H­(FNA)), 8.56 (2 H, br, NH­(Ala 2), NH­(Cys)), 8.78 (1 H, d, 2-H­(FNA)). δ ^13^C (125 MHz; DMSO-*d*6) 17.06 Me­(Ala1), 17.74 Me­(Val), 17.94 Me­(Ala2), 19.02 Me­(Leu), 21.44 Me­(Val), 22.85 Me­(Leu), 23.95 Me­(Leu), 23.97 Me­(Leuγ), 30.46 Cβ­(Val), 30.81 Cβ­(Cys), 40.72 Cβ­(Leu 1,2), 41.51 Cα­(Gly1), 41.95 Cα­(Gly2, 3), 47.79 Cα­(Ala2), 48.08 Cα­(Ala1), 50.91 Cα­(Leu 2), 51.36 Cα­(Cys), 51.75 Cα­(Leu2), 55.14 Cα­(Ser), 57.42 Cα­(Val), 61.59 Cβ­(Ser), 110.25 (d, 5-C­(FNA)), 130.9 (d, 3-C­(FNA)), 140.94 (d, 4-C­(FNA)), 147.05 (d, 2-C­(FNA)), 164.71 (d, 6-C­(FNA)), 167.94 CO­(Gly1), 167.93 CO­(Gly2), 168.54 CO­(Gly3), 169.30 CO­(Cys), 169.62 CO­(Ala1), 170.18 CO­(Ser), 170.30 CO­(Val), 171.73 CO­(Leu2), 172.27 CO­(Leu1), 174.01 CO­(Ala2), 188.16 CO­(FNA). MS *m*/*z*: [M + H]^+^ calculated for C_41_H_66_FN_12_O_12_S, 969.4622; found 969.4615.

### Preparation of FNA-*S*-C@5

Part of the experimental procedures and chemical characterization data have been described in an academic thesis of Master of Science degree at the University of Turku, Finland.^20^ Similarly, compound FNA-*S*-C@5 (1.1 mg, 1.1 μmol) was prepared as a white solid product in 18.6% isolated chemical yield, and the retention time was 6.2 min using semipreparative HPLC. The chemical structure of FNA-*S*-C@5 was characterized as follows. δ ^1^H (600 MHz; DMSO-*d*6) 0.80 (3 H, d, Me­(Val)), 0.82 (3 H, d, Me­(Leu)), 0.83 (3 H, d, Me­(Leu)), 0.84 (3 H, br, Me­(Val)), 0.86 (6 H, d, Me­(Leu)), 1.23 (3 H, dd, Me­(Ala2)), 1.35 (3 H, br, Me­(Ala1)), 1.45 (4 H, m, CH_2_(Leu 1, 2β)), 1.58 (2 H, m, CH­(Leuγ)), 1.97 (1 H, m, CH­(Valβ)), 3.31 (1 H, br, CH_2_(Cysβ)), 3.56 (1 H, d, CH_2_(Cysβ)), 3.59 (1 H, m, CH_2_(Serβ)), 3.64­(1 H, br, CH­(Ala1α)), 3.66 (1 H, m, CH_2_(Serβ)), 3.73–3.81 (6 H, d, CH­(Glyα)), 4.15 (1 H, m, CH­(Valα)), 4.18 (1 H, m, CH­(Ala2α)), 4.28 (1 H, m, CH­(Serα)), 4.30 (1 H, m, CH­(Leu2α)), 4.38 (1 H, q, CH­(Leu1α)), 4.56 (1 H, q, CH­(Cysα)), 5.05 (1 H, br, SerOH), 6.96 (1 H, s, CONH_2_(Ala2)), 7.20 (1 H, s, CONH_2_(Ala2)), 7.39 (1 H, dd, 5-H­(FNA)), 7.74 (1 H, d, NH­(Ala 2)), 8.00 (2 H, d, NH­(Val), NH­(Leu1)), 8.05 (1H, d, NH­(Ala1)), 8.06 (1 H, d, NH­(Leu2)), 8.11­(1 H, d, NH­(Ser)), 8.23 (1 H, m, NH­(Cys)), 1 H, m, NH­(Gly2)), 8.26 (1 H, t, NH­(Gly1)), 8.37 (1 H, br, NH­(Gly3)), 8.43­(1 H, dt, 4-H­(FNA)), 8.75 (1 H, d, 2-H­(FNA)). δ ^13^C (125 MHz; DMSO-*d*6)­17.07 Me­(Ala1), 17.66 Me­(Val), 18.94 Me­(Val), 21.35 Me­(Leu), 22.79 Me­(Leu), 22.83 Me­(Leu), 23.77 Me­(Leuγ), 28.53 Me­(Ala2), 30.36 Cβ­(Val), 30.65 Cβ­(Cys), 40.72 Cβ­(Leu1, Leu2), 41.59 Cα­(Gly), 41.82 Cα­(Gly), 47.75 Cα­(Ala2), 50.57 Cα­(Leu2), 50.80 Cα­(Leu2), 51.36 Cα­(Cys), 54.80 Cα­(Ser), 57.34 Cα­(Val), 60.99 Cβ­(Ser), 110.24 (d, 5-C­(FNA)), 131.70 (d, 6-C­(FNA)), 131.89 (d, 3-C­(FNA)), 140.87 (d, 4-C­(FNA)), 146.58 (d, 2-C­(FNA)), 169.16 CO­(Gly), 169.58 CO­(Gly), 168.93 CO­(Gly), 168.84 CO­(Cys), 169.57 CO­(Ser), 169.73 CO­(Ala2), 171.33 CO­(Leu1), 171.54 CO­(Leu2), 173.12 CO­(Ala1), 188.97 CO­(FNA). MS *m*/*z*: [M + H]^+^ calculated for C_41_H_66_FN_12_O_12_S, 969.4622; found 969.4623.

### Peptide Conjugation Tests Using [^18^F]­FNA 4-Nitrophenyl Ester at pH 8.6 and 7.4

[^18^F]­FNA 4-nitrophenyl ester was prepared similarly as previously published.^10,11^ In the conjugation tests, 10 μL of [^18^F]­FNA (approximately 10 MBq) in acetonitrile was diluted with 300 μL of acetonitrile, and 5.0 mg (5.9 μmol) of peptide (C@3, C@4, or C@5) in 350 μL of borate buffer (300 mM, pH 8.6) was added. The reaction mixture was kept at r.t. for 10 min, and a sample was taken for analytical HPLC analysis. In each case, the chemical identity of the conjugation product was confirmed by coinjection of a nonradioactive reference compound with the radioactive samples. HPLC analysis was performed on a reversed-phase C18 column (Phenomenex, 250 × 4.6 mm, particle size 4 μm, and pore size 90 Å). The solvent flow rate was 1 mL/min. Solutions A and B were prepared using 0.1% TFA in water and 0.1% TFA in acetonitrile, respectively. The gradient method used for the analysis was recorded as 0–12 min from 20% B to 50% B and 12–16 min from 50% B to 80% B. HPLC detection methods were with both radioactivity detection and UV detection at a wavelength of 220 nm. Similarly, peptide conjugation tests were performed using phosphate buffer (300 mM) at pH 7.4.

### Radiosynthesis of [^18^F]­FNA-*S*-C@5

To demonstrate the chemoselective *S*-acylation for radiosynthesis with a whole batch of the prosthetic compound [^18^F]­FNA 4-nitrophenyl ester, peptide C@5 was used as an example (Figure 7). Radiosynthesis was performed using a custom-made device (DM Automation, Nykvarn, Sweden).^21^ Liquids are carried by pressurized nitrogen gas through the microcapillaries of the synthesis apparatus. Our in-house–produced [^18^F]­fluoride (7.5 ± 1.2 GBq, n = 3) was run through a Sep-Pak Accell Plus QMA Plus Light cartridge, which retained the fluoride. The [^18^F]­fluoride was eluted from the cartridge with a mixture of Kryptofix 222 and potassium carbonate in acetonitrile and water,^21^ and transferred to the reaction vessel. Heating the reaction vessel to 120 °C for 15 min with a continuous flow of nitrogen gas removed the residual water from the [^18^F]­fluoride solution. The vessel temperature was then decreased to 37 °C. The primary precursor *N,N,N-*trimethyl-5-((4-nitrophenoxy)­carbonyl)­pyridin-2-aminium trifluoromethanesulfonate (8.0 mg, 0.02 mmol) was immersed in 1,4-diazabicyclo[2.2.2]­octane (14 mg, 0.13 mmol) in a mixture of acetonitrile and *tert*-butanol (1:4, v/v, 1.2 mL), placed in the reaction vessel, and allowed to react for 10 min at 37 °C. The reaction mixture was diluted with water (100 μL) for subsequent purification on a reversed-phase C18 HPLC column (Phenomenex, Jupiter Proteo 4 μm, 250 × 10 mm, 90 Å). The mobile phase consisted of Milli-Q water (Solvent A) and acetonitrile (Solvent B), each containing 0.1% TFA. A Hitachi L-6200 pump was used to transport the mobile phase. The HPLC elution program was 45% of solvent B during 0–13 min and 45%–70% of solvent B during 13–25 min. At 15 min, the [^18^F]­FNA 4-nitrophenyl ester was detected and collected. The purified product from the [^18^F]­FNA 4-nitrophenyl ester HPLC fraction was extracted using an HLB cartridge (30 mg, Waters, Milford, MA, USA). Thereafter, the cartridge was rinsed with 5 mL of water, and 0.4 mL of acetonitrile was used to extract the [^18^F]­FNA 4-nitrophenyl ester from the cartridge into a separate reaction vessel. The C@5 peptide (5.0 mg, 5.9 μmol) was amalgamated with 450 μL of borate buffer (pH 8.6, 300 mM) and 150 μL of water in the reaction vessel to synthesize [^18^F]­FNA*-S-*C@5 for 10 min at r.t. The reaction mixture was subjected to HPLC purification as described above, except that the HPLC gradient program was 0% of solvent B during 0–5 min and 0%–55% of solvent B during 5–20 min. [^18^F]­FNA-*S*-C@5 appeared at approximately 25 min, and the HPLC fraction was collected in a container.

### In Vitro Stability Tests in Rat Plasma

The in vitro stability of [^18^F]­FNA-*S*-C@5 was assessed in plasma from male Sprague–Dawley rats similarly as a previously published procedure.^8^ Accordingly, [^18^F]­FNA-*S*-C@5 (0.2 MBq) was incubated in 0.4 mL of plasma at 37 °C, and samples of 65 μL were taken at 5 and 15 min of incubation. To precipitate plasma proteins, 70 μL of acetonitrile was added to each sample, followed by centrifugation at 12,000 × *g* for 2 min. The supernatant was then injected into a reversed-phase C18 column (Jupiter Proteo, 250 × 10 mm, 4 μm, 90 Å; Phenomenex) for HPLC analysis at a flow rate of 5 mL/min, with radioactivity and UV detection at 220 nm. The HPLC solvent A was 0.1% TFA in water, and solvent B was 0.1% TFA in acetonitrile. The elution gradient ranged from 15% to 55% B over 0 to 16 min. The experiments were performed in triplicate for both time points. The presence of intact [^18^F]­FNA-*S*-C@5 among the radiometabolites in the plasma samples was verified using a reference standard of [^18^F]­FNA-*S*-C@5.

## Supplementary Material



## Notes

Detailed method for LC-ESI-MS/MS analysis and NMR spectra of peptides (Figures S1–S13) (PDF)

## Abbreviations

ALARA: as low as reasonably achievable
DMSO: dimethyl sulfoxide
^18^F: fluorine-18
[^18^F]­FNA: fluorine-18-labeled 6-fluoronicotinic acid
FNA: 6-fluoronicotinic acid
HLB: hydrophilic–lipophilic balanced
HMBC: heteronuclear multiple bond correlation
HPLC: high-performance liquid chromatography
HSQC: heteronuclear single quantum coherence
LC-ESI-MS/MS: liquid chromatography-electrospray ionization-mass spectrometry/mass spectrometry
NMR: nuclear magnetic resonance
PET: positron emission tomography
r.t.: room temperature
TFA: trifluoroacetic acid
TOCSY: total correlation spectroscopy
UV: ultraviolet
1D: one-dimensional
2D: two-dimensional
