# Supplementary material for: Exclusively Chemoselective S‑Acylation for Peptide Radiolabeling Using [18F]Fluoronicotinic Acid 4‑Nitrophenyl Ester as a Prosthetic Compound
Source: ACS Omega. 2026 Jun 11;11(24):35280–90. doi: 10.1021/acsomega.6c00097 (PMC13294875; doi:10.1021/acsomega.6c00097)
Supplement: Supplementary file 1 [file ao6c00097_si_001.pdf]

Supporting Information

Exclusively chemoselective *S*-acylation for peptide radiolabeling using [^{18}F]fluoronicotinic acid 4-nitrophenyl ester as a prosthetic compound

Nelson Nwaenie,[a,b] *Tuomas Karskela,*[c] *Pyry Dillemuth*[a,b] *Johan Rajander,*[a,d] *Pirjo Laakkonen,*[e,f,g] *Anu J. Airaksinen,*[a,b,h] *Xiang-Guo Li*[a,b,h,i*]

[a]Turku PET Centre, University of Turku, Kiinamyllynkatu 4-8, FI-20520 Turku, Finland

[b]Department of Chemistry, University of Turku, Henrikinkatu 2, FI-20500 Turku, Finland

[c]Turku Centre for Chemical and Molecular Analytics (CCMA), Åbo Akademi University, FI-20500 Turku, Finland

[d]Accelerator Laboratory, Åbo Akademi University, Kiinamyllynkatu 4-8, FI-20520 Turku, Finland

[e]Translational Cancer Medicine Research Program, Faculty of Medicine, University of Helsinki, Haartmaninkatu 8, FI-00014 Helsinki, Finland

[f]iCAN Flagship Program, University of Helsinki, Haartmaninkatu 8, FI-00014 Helsinki, Finland

[g]Laboratory Animal Centre, HiLIFE University of Helsinki, Haartmaninkatu 8, FI-00014 Helsinki, Finland

[h]Turku PET Centre, Turku University Hospital, Kiinamyllynkatu 4-8, FI-20520 Turku, Finland

[i]InFLAMES Research Flagship, University of Turku, Tykistökatu 6, FI-20520 Turku, Finland

***Corresponding Author:** Associate Professor Xiang-Guo Li, Turku PET Centre, University of Turku, Kiinamyllynkatu 4-8, FI-20520 Turku, Finland. Phone: +358 50 4485069; E-mail: xiali@utu.fi

1. Liquid Chromatography-Electrospray Ionization-Mass Spectrometry Analysis

The liquid chromatography-electrospray ionization-mass spectrometry (LC-ESI-MS)/MS analysis was performed on a nanoflow HPLC system (Easy-nLC1000, Thermo Fisher Scientific) coupled to a Q Exactive HF mass spectrometer (Thermo Fisher Scientific) equipped with a nano-electrospray ion source. First, the sample was loaded onto a trapping column (100 μm ID × 2 cm) and subsequently separated inline on an analytical column (75 μm ID × 15 cm). The mobile phase consisted of water with 0.1% formic acid (Solvent A) and acetonitrile/water (80:20 (v/v)) with 0.1% formic acid (Solvent B). Peptides were eluted with the following gradient: from 10% to 50% of Solvent B in 10 min, from 50% to 100% of Solvent B in 5 min, and then washed for 5 min at 100% of Solvent B. Thermo Xcalibur 4.1 software (Thermo Fisher Scientific) was used to automatically acquire MS data. The data-dependent acquisition method consisted of repeated cycles of MS1 scan covering a range of m/z 300–2,000, followed by Higher-energy Collisional Dissociation (HCD) fragment ion scans (MS2 scans) for up to six of the most intense precursor ions from the MS1 scan. Stepped collision energies (Normalized Collison Energy (NCE) 15%, NCE 20%, and NCE 25%) were used in HCD fragmentation.

2. NMR Spectra

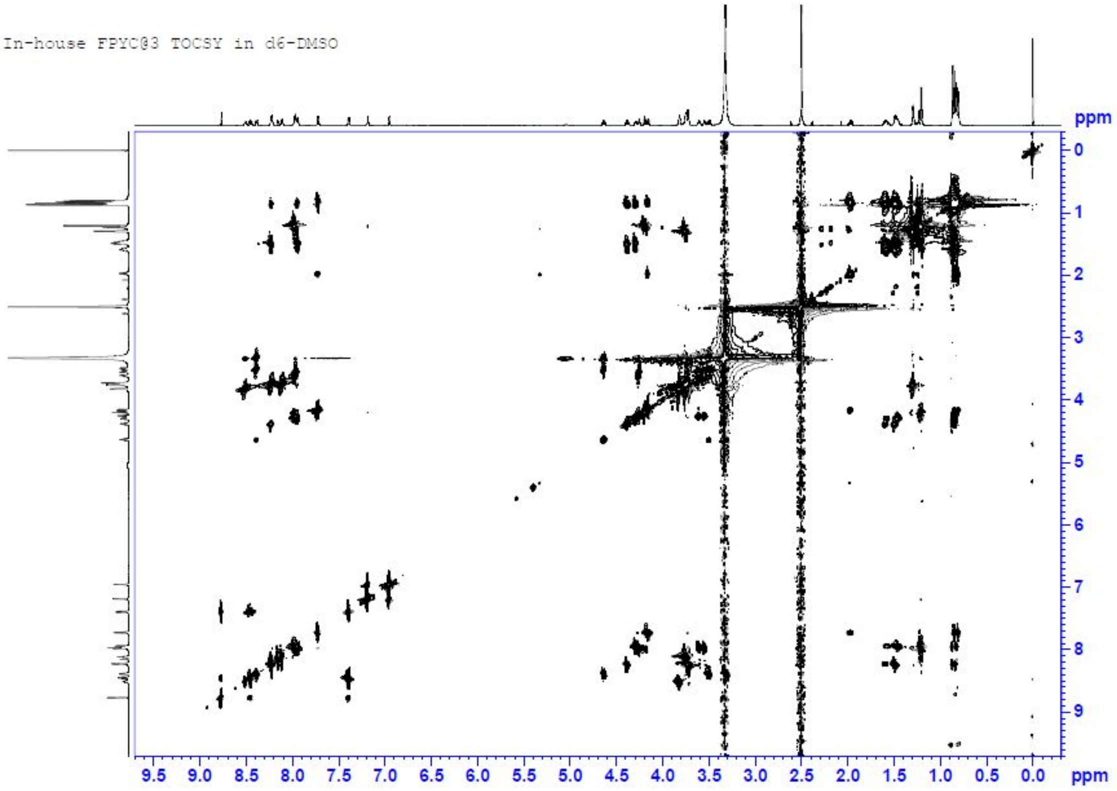

Figure S1. TOCSY spectrum of FNA-*S*-C@3.

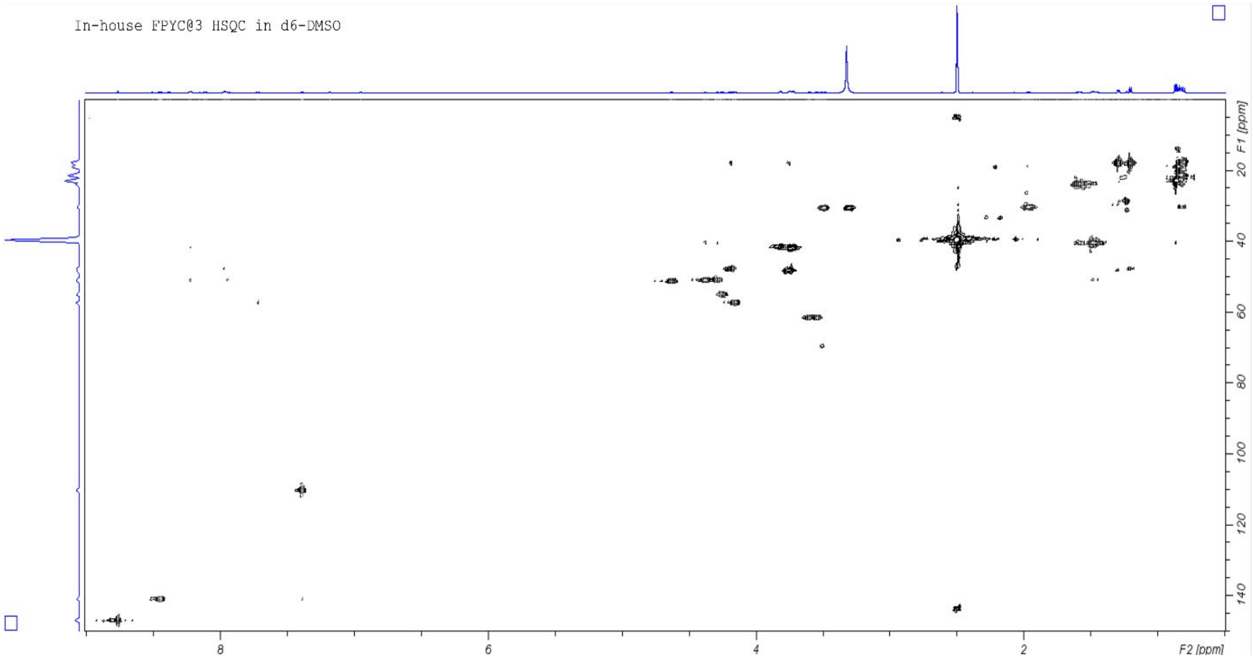

Figure S2. HSQC spectrum of FNA-*S*-C@3.

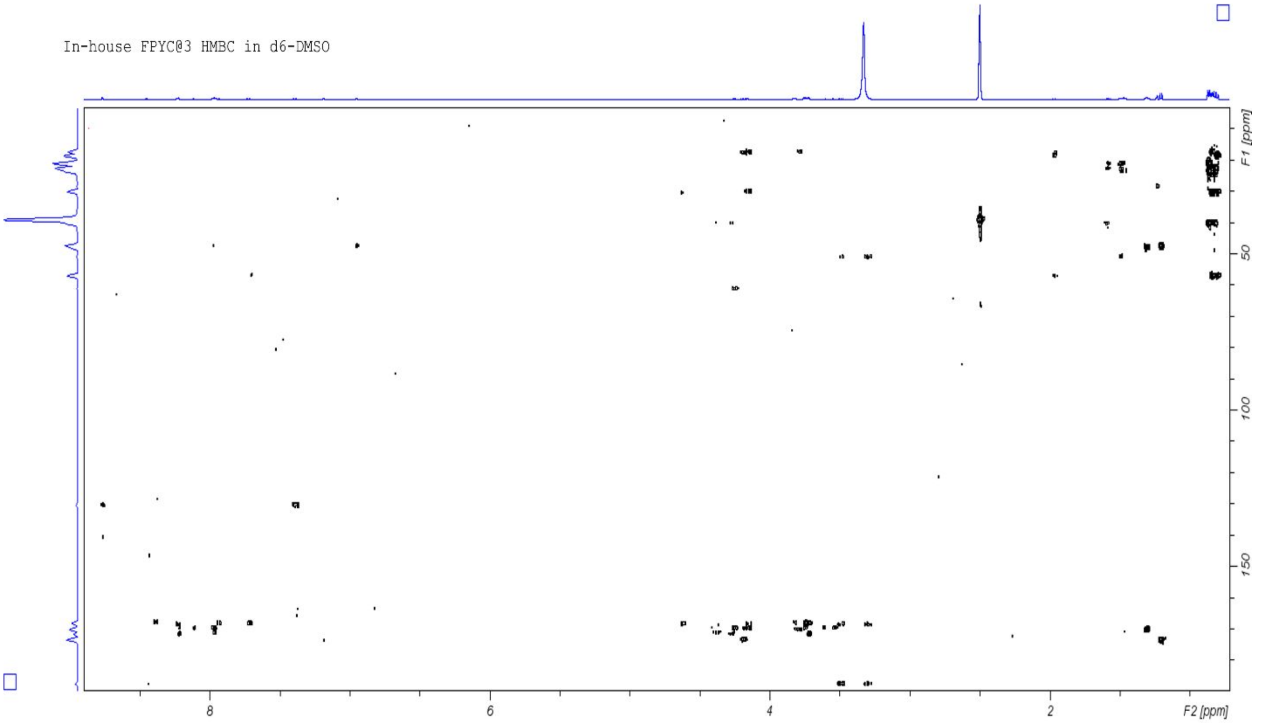

Figure S3. HMBC spectrum of FNA-*S*-C@3.

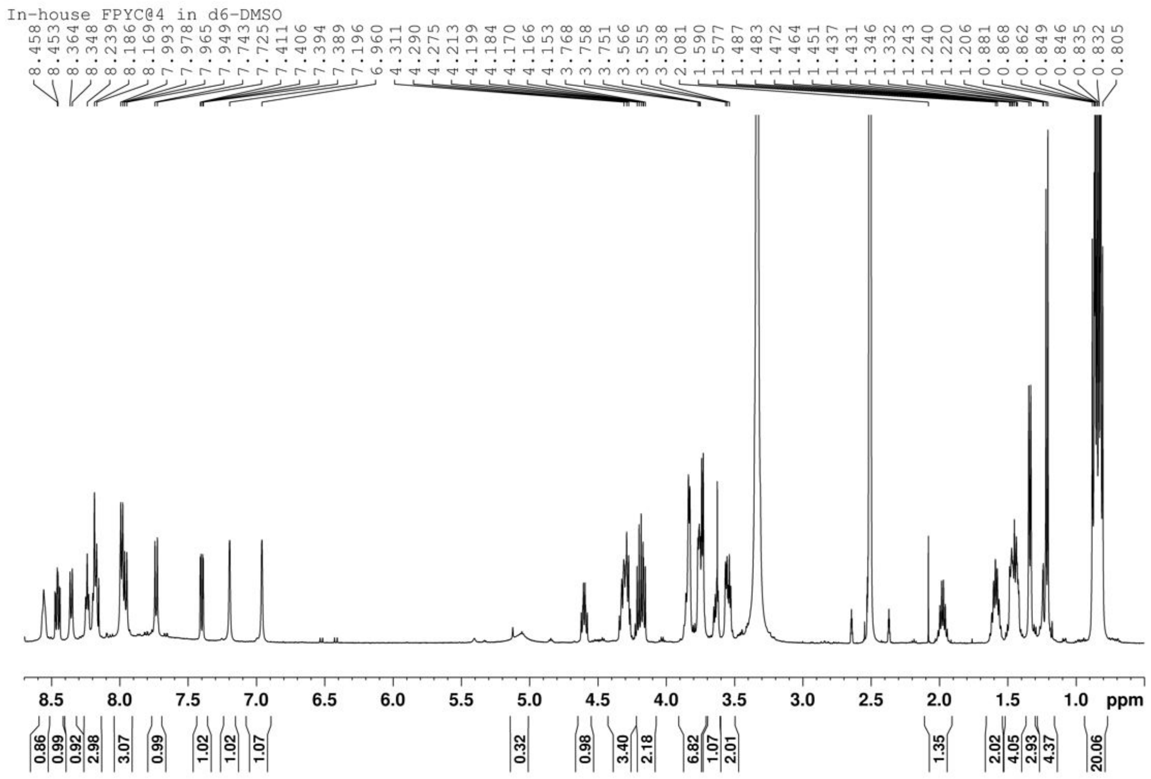

Figure S4. ^{1}H-NMR spectrum of FNA-*S*-C@4.

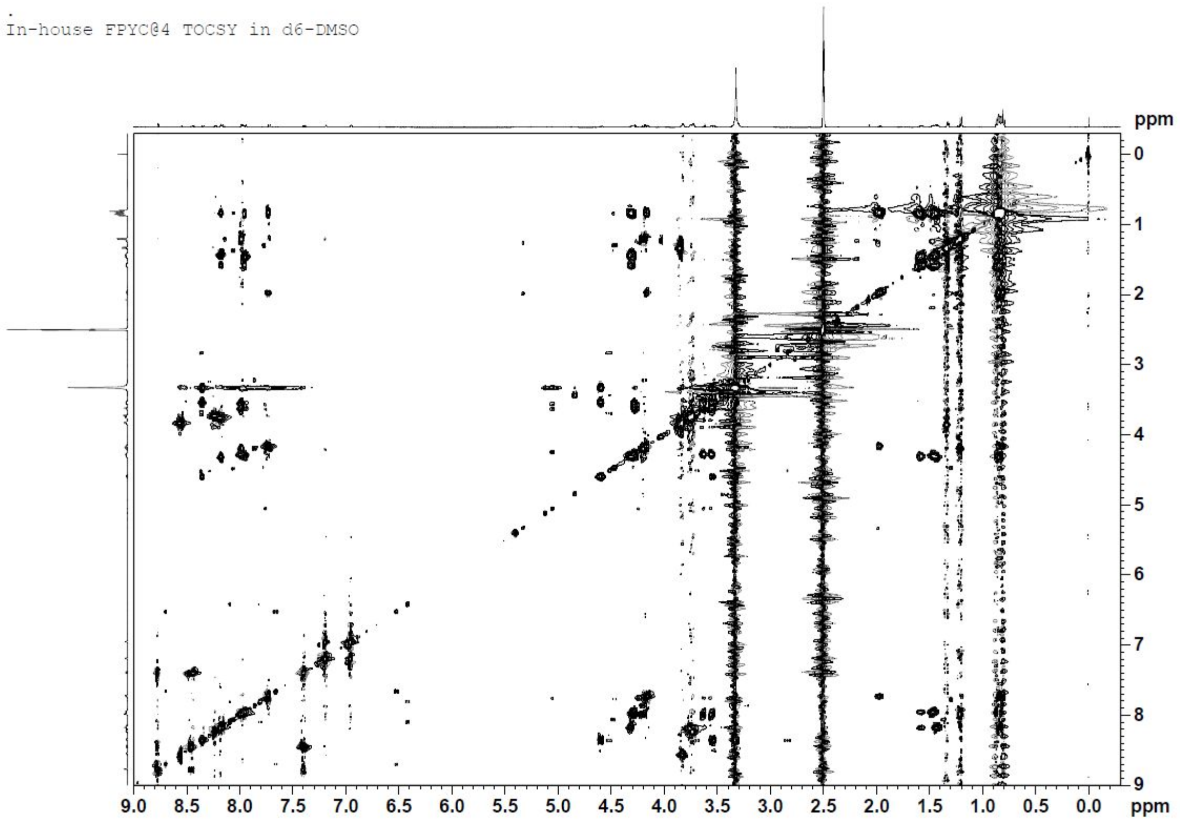

Figure S5. TOCSY spectrum of FNA-*S*-C@4.

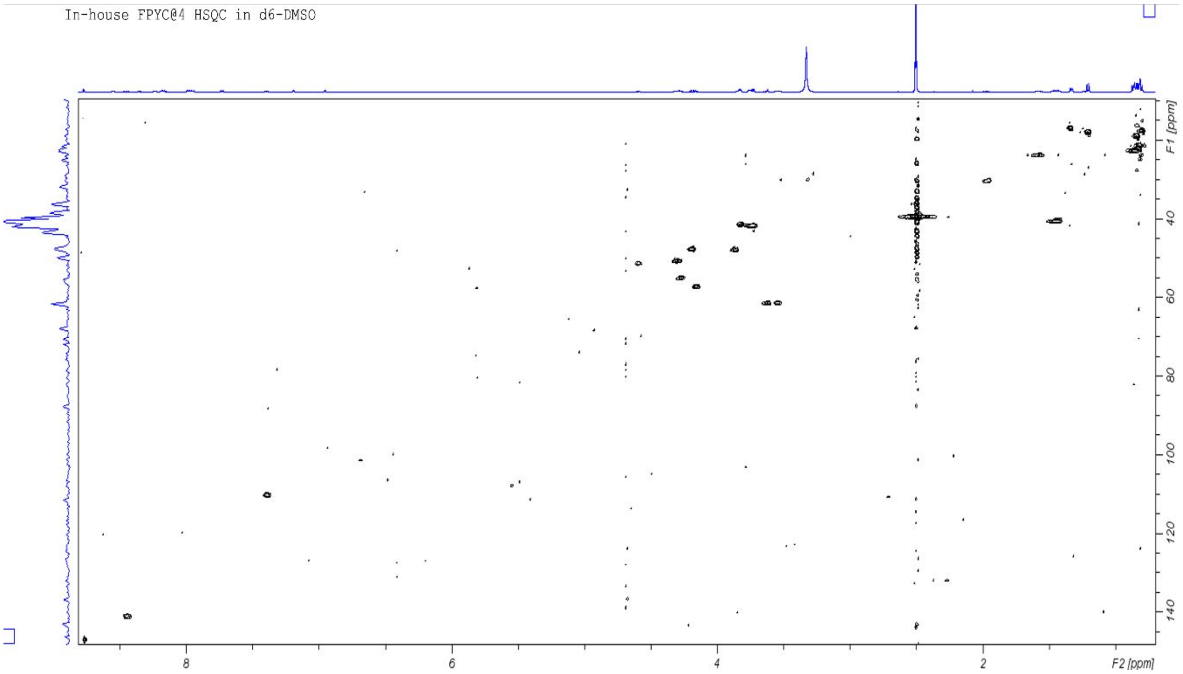

Figure S6. HSQC spectrum of FNA-*S*-C@4.

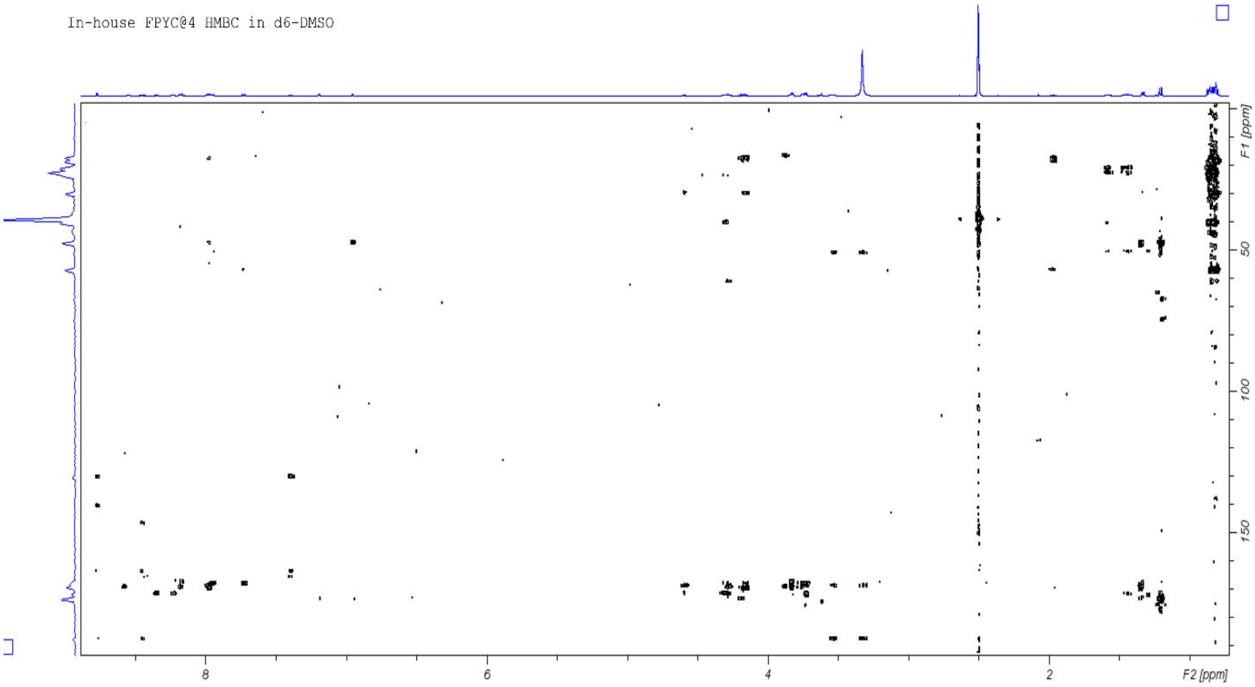

Figure S7. HMBC spectrum of FNA-*S*-C@4.

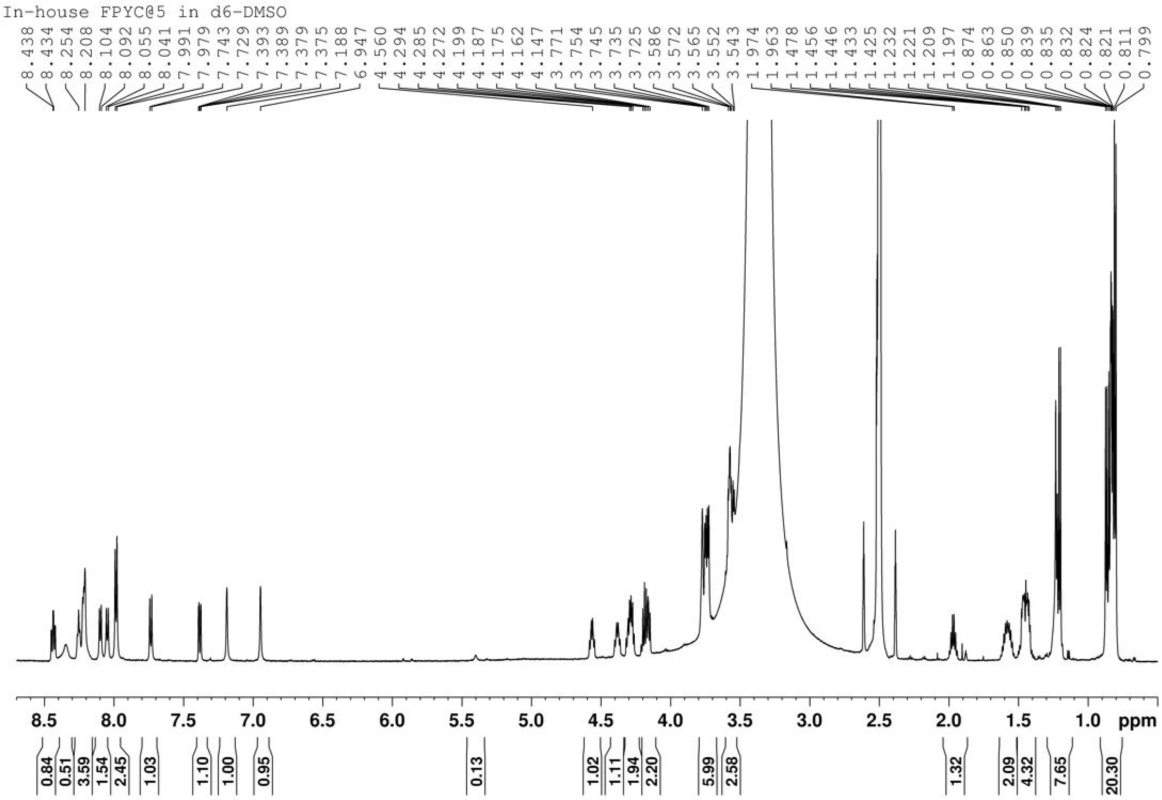

Figure S8. ^{1}H-NMR spectrum of FNA-*S*-C@5.

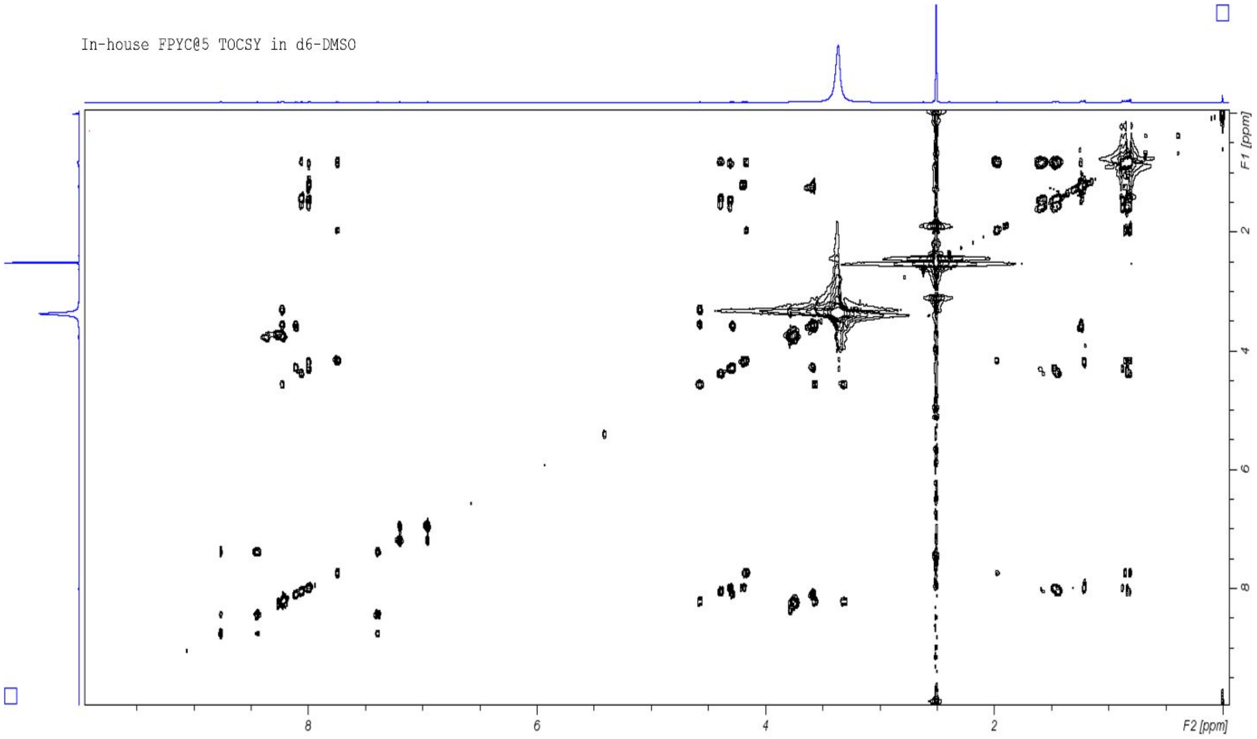

Figure S9. TOCSY spectrum of FNA-*S*-C@5.

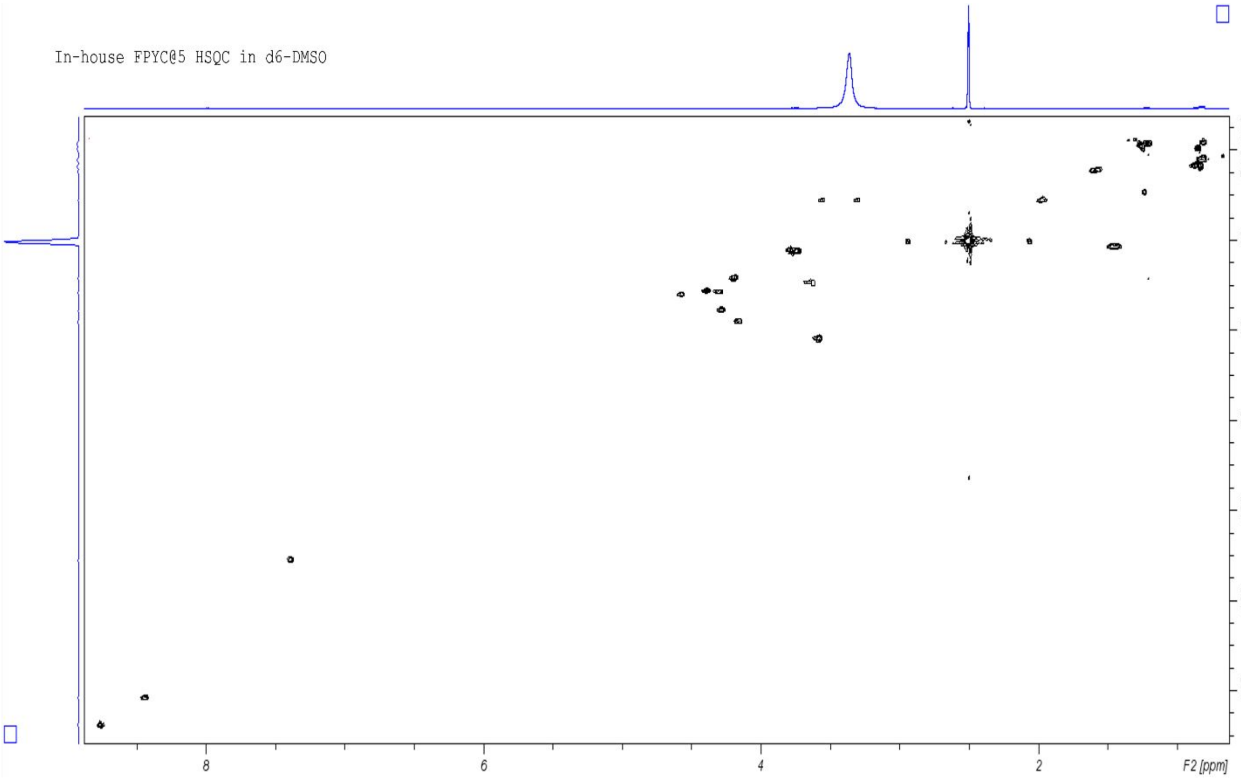

Figure S10. HSQC spectrum of FNA-*S*-C@5.

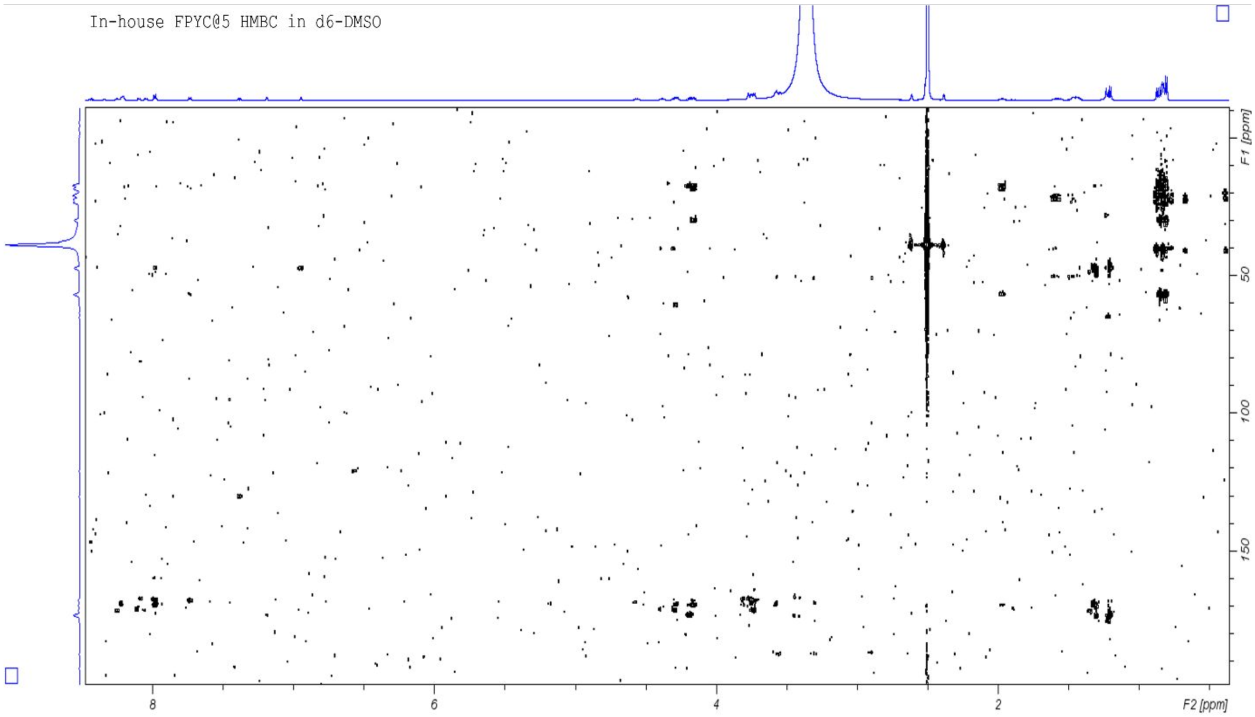

Figure S11. HMBC spectrum of FNA-*S*-C@5.

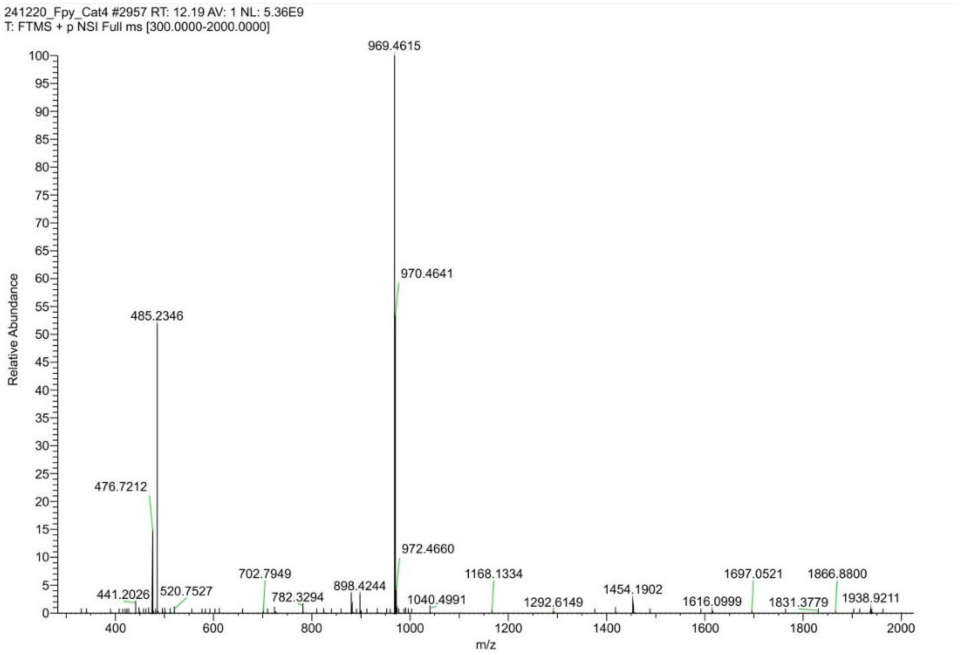

Figure S12. MS spectrum of FNA-*S*-C@4.

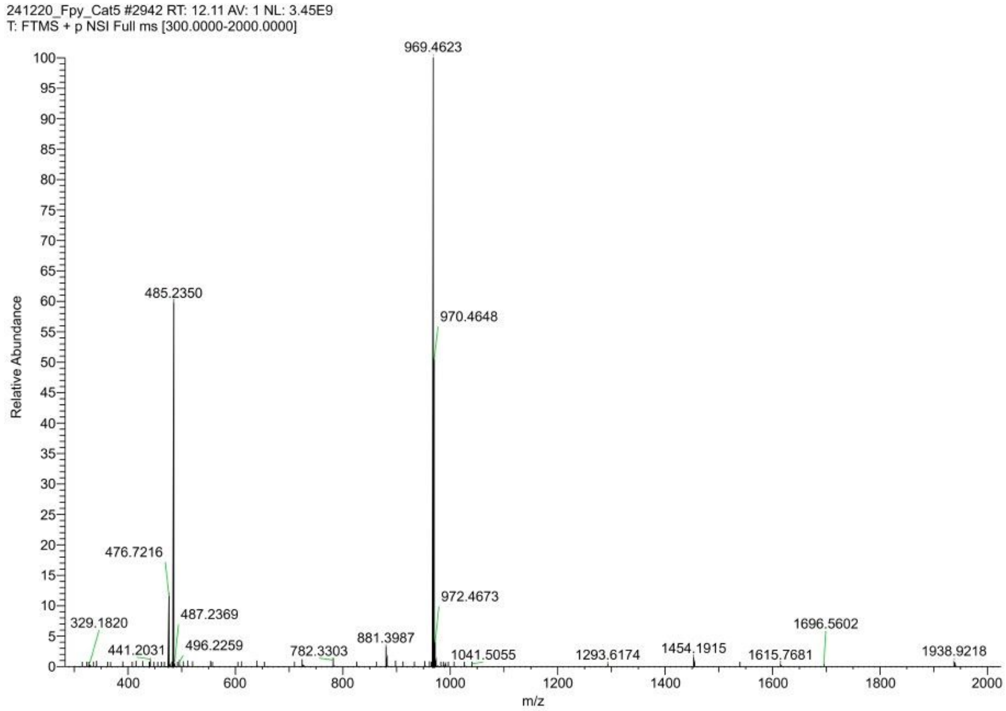

Figure S13. MS spectrum of FNA-*S*-C@5.

Table S1. Reaction conditions and conjugation products in experiments with and without radioactivity (All reactions were performed at pH 8.6 for 10 min at room temperature).

Peptide (Concentration)	Prosthetic compound (Concentration)	Product	Side products
C@3 (14 mM)	[^{18}F]FNA (< 20 μM)	>95% of [^{18}F]FNA-*S*-C@3*	< 5% of unidentified compounds
	FNA (44 mM)	FNA-*S*-C@3 in 15.3% isolated yield#	Several unidentified compounds
C@4 (14 mM)	[^{18}F]FNA (< 20 μM)	>99% of [^{18}F]FNA-*S*-C@4*	No side product was observed.
	FNA (44 mM)	FNA-*S*-C@4 in 16.9% isolated yield#	Several unidentified compounds
C@5 (14 mM)	[^{18}F]FNA (< 20 μM)	>95% of [^{18}F]FNA-*S*-C@5*	< 5% of unidentified compounds
	FNA (44 mM)	FNA-*S*-C@5 in 18.6% isolated yield#	Several unidentified compounds

*This was calculated from HPLC analysis from the reaction mixture without any purification. #This was the isolated yield. The chemical identity of other possible products originated from peptide conjugation was not confirmed, which did not allow to determine the conversion of the desired product in the samples from reaction mixtures. The chemical identity of the side products was not confirmed. Therefore, we cannot conclude that the reactions were chemoselective toward *S*-acylation in these nonradioactive conjugation experimental settings.
